# Artificial Neural Networks Predicted the Overall Survival and Molecular Subtypes of Diffuse Large B-Cell Lymphoma Using a Pancancer Immune-Oncology Panel

**DOI:** 10.3390/cancers13246384

**Published:** 2021-12-20

**Authors:** Joaquim Carreras, Shinichiro Hiraiwa, Yara Yukie Kikuti, Masashi Miyaoka, Sakura Tomita, Haruka Ikoma, Atsushi Ito, Yusuke Kondo, Giovanna Roncador, Juan F. Garcia, Kiyoshi Ando, Rifat Hamoudi, Naoya Nakamura

**Affiliations:** 1 Department of Pathology, School of Medicine, Tokai University, 143 Shimokasuya, Isehara 259-1193, Japan; hiraiwa19@tokai-u.jp (S.H.); ki285273@tsc.u-tokai.ac.jp (Y.Y.K.); mm946645@tsc.u-tokai.ac.jp (M.M.); hs800759@tsc.u-tokai.ac.jp (S.T.); oh298955@tsc.u-tokai.ac.jp (H.I.); ito.atsushi.s@tokai.ac.jp (A.I.); kondou@tokai-u.jp (Y.K.); naoya@is.icc.u-tokai.ac.jp (N.N.); 2 Monoclonal Antibodies Unit, Spanish National Cancer Research Center (Centro Nacional de Investigaciones Oncologicas, CNIO), Melchor Fernandez Almagro 3, 28029 Madrid, Spain; groncador@cnio.es; 3 Department of Pathology, MD Anderson Cancer Center Madrid, Calle de Arturo Soria 270, 28033 Madrid, Spain; jfgarcia@mdanderson.es; 4 Department of Hematology, School of Medicine, Tokai University, 143 Shimokasuya, Isehara 259-1193, Japan; andok@keyaki.cc.u-tokai.ac.jp; 5 Sharjah Institute for Medical Research, Department of Clinical Sciences, College of Medicine, University of Sharjah, Sharjah P.O. Box 27272, United Arab Emirates; rhamoudi@sharjah.ac.ae; 6 Division of Surgery and Interventional Science, University College London, Gower Street, London WC1E 6BT, UK

**Keywords:** artificial intelligence, artificial neural networks, multilayer perceptron, radial basis function, machine learning, diffuse large B-cell lymphoma, prognosis, overall survival, molecular subtype, pancancer immune-oncology panel

## Abstract

**Simple Summary:**

This research predicted the overall survival of patients and cell-of-origin molecular subtypes of diffuse large B-cell lymphoma from Tokai University using gene expression data. A pancancer immune profiling panel was analyzed using artificial neural networks, and high accuracy of prediction was found. Additionally, the results were explained with other machine learning techniques and conventional bioinformatics analyses.

**Abstract:**

Diffuse large B-cell lymphoma (DLBCL) is one of the most frequent subtypes of non-Hodgkin lymphomas. We used artificial neural networks (multilayer perceptron and radial basis function), machine learning, and conventional bioinformatics to predict the overall survival and molecular subtypes of DLBCL. The series included 106 cases and 730 genes of a pancancer immune-oncology panel (nCounter) as predictors. The multilayer perceptron predicted the outcome with high accuracy, with an area under the curve (AUC) of 0.98, and ranked all the genes according to their importance. In a multivariate analysis, *ARG1*, *TNFSF12*, *REL*, and *NRP1* correlated with favorable survival (hazard risks: 0.3–0.5), and *IFNA8*, *CASP1*, and *CTSG*, with poor survival (hazard risks = 1.0–2.1). Gene set enrichment analysis (GSEA) showed enrichment toward poor prognosis. These high-risk genes were also associated with the gene expression of M2-like tumor-associated macrophages (*CD163*), and *MYD88* expression. The prognostic relevance of this set of 7 genes was also confirmed within the IPI and *MYC* translocation strata, the EBER-negative cases, the DLBCL not-otherwise specified (NOS) (High-grade B-cell lymphoma with *MYC* and *BCL2* and/or *BCL6* rearrangements excluded), and an independent series of 414 cases of DLBCL in Europe and North America (GSE10846). The perceptron analysis also predicted molecular subtypes (based on the Lymph2Cx assay) with high accuracy (AUC = 1). *STAT6*, *TREM2*, and *REL* were associated with the germinal center B-cell (GCB) subtype, and *CD37*, *GNLY*, *CD46*, and *IL17B* were associated with the activated B-cell (ABC)/unspecified subtype. The GSEA had a sinusoidal-like plot with association to both molecular subtypes, and immunohistochemistry analysis confirmed the correlation of *MAPK3* with the GCB subtype in another series of 96 cases (notably, MAPK3 also correlated with LMO2, but not with M2-like tumor-associated macrophage markers CD163, CSF1R, TNFAIP8, CASP8, PD-L1, PTX3, and IL-10). Finally, survival and molecular subtypes were successfully modeled using other machine learning techniques including logistic regression, discriminant analysis, SVM, CHAID, C5, C&R trees, KNN algorithm, and Bayesian network. In conclusion, prognoses and molecular subtypes were predicted with high accuracy using neural networks, and relevant genes were highlighted.

## 1. Introduction

Diffuse large B-cell lymphoma (DLBCL) is one of the most frequent non-Hodgkin lymphomas (NHL) in developed Western and Asian countries, representing around 25% of NHL cases [1,2,3].

DLBCL is a heterogeneous entity because of its diverse histological and genetic features and clinical evolution. There are several subtypes of DLBCL, such as T cell/histiocyte-rich large B-cell lymphoma, primary DLBCL of the mediastinum, intravascular large B-cell lymphoma, primary DLBCL of the central nervous system, Epstein–Barr virus (EBV)-positive DLBCL, etc. Additionally, some cases overlap with Burkitt lymphoma and were previously referred as “Burkitt-like”. Currently, the term High-grade B-cell lymphoma with *MYC* and *BCL2* and/or *BCL6* rearrangements is used [1,2].

With current rituximab-based therapy, DLBCL is curable in around 50% of cases [4]. Therefore, at diagnosis, it is important to identify and predict which patients will clinically evolve unfavorably. The prognosis of DLBCL can be assessed with several variables, such as the International Prognostic Index (IPI), which includes several clinical and biochemical variables (age, LDH, ECOG performance status, clinical stage, and extranodal sites); cell of origin molecular subtypes (gene expression profiling, Hans, Choi, and Tally algorithms, and the Lymph2Cx platform) [5,6,7,8,9]; *MYC*, *BCL2*, and *BCL6* abnormalities; and the tumor immune microenvironment [10,11,12,13]. Based on gene expression, three types of DLBCL have been defined: germinal center B-cell-like (GCB), activated B-cell-like (ABC), and not-otherwise-specified type 3 (i.e., unclassified, unspecified).

It is recommended that all cases undergo assessment of the molecular subtype at diagnosis. The gold standard is gene expression profiling (GEP) using the “lymphochip” microarray, but this technique requires the use of frozen tissue, which is not always available. Currently, the molecular subtype can be assessed using formalin-fixed paraffin-embedded tissue (FFPET) samples using the nCounter NanoString platform [8]. This array uses the gene expression of 32 genes, including the known markers of Hans’ classifier *MME* (CD10), *BCL6*, and *IRF4* (MUM-1), the *LMO2* gene of the Tally algorithm, and other relevant pathogenic genes such as *BCL2*, *BTK*, *CARD11*, *MYD88*, and *TP53*. Interestingly, the genes *GCET1* and *FOXP1* of the Choi algorithm are excluded in this panel.

The immuno-oncology pathway is now important in the analysis of the pathogenesis of DLBCL because through it, actionable gene expression profiles in the context of cancer immunotherapy can be identified. The nCounter pancancer immune profiling panel performs multiplex gene expression analysis in humans with 770 genes (40 housekeeping and 730 immune oncology genes) from different immune cell types, common checkpoint inhibitors, CT antigens, and genes covering both adaptive and innate immune response [14].

Some of the most impressive recent advances in artificial intelligence (AI) have been in the field of deep learning [15]. Deep learning models have neared or even exceeded human-level performance [15]. Artificial neural networks (ANNs) are a set of algorithms that were designed based on the human brain to identify patterns [11,12,16]. ANNs interpret sensory data through a kind of machine perception, labeling or clustering of raw input data/information [11,12,16]. The patterns that ANNs recognize are numerical, contained in vectors into which all real-world data (be they images, sound, text, or time series) must be translated [11]. As an approach to machine learning, ANNs can handle complex patterns found in the most challenging real-word datasets. ANNs use nonlinear modeling to identify complex relationships between variables and to create predictive models. ANNs provide an alternative predictive capability to approaches such as regression and classification trees and are characterized by being flexible and the lack of distributional assumptions [17,18]. In predictive applications, the multilayer perceptron (MLP) and the radial basis function (RBF) networks are commonly used. Both networks are supervised, because the results can be compared against known values of the target variables [10,11,12,17,18]. Both MLP and RBF have a “feedforward architecture”, because the connections in the network flow from the input layer to the output layer without any feedback loops. The architecture comprises the following parts: (1) an input layer that contains the predictors; (2) a hidden layer with unobservable nodes, or units; and (3) the output layer that contains the responses. The value of each hidden unit is some function of the predictors. The choice between MLP and RBF is influenced by the type of data to be analyzed and the level of complexity to uncover. Generally, the MLP procedure can handle more complex relationships. Conversely, the RBF procedure, which is characterized by one hidden layer, is usually faster [10,11,12,13,17,18,19,20,21].

Explainable AI (XAI) is attracting much interest in medicine [22]. XAI deals with the implementation of transparency and traceability of statistical black-box machine learning methods, particularly deep learning [22]. In the machine-based decision-making process, it is crucial to reproduce and comprehend both the learning and knowledge-extraction processes [22,23]. This is important, because for decision support it is necessary to understand the causality of learned representations [22,23]. In this research, machine learning techniques and conventional statistics were performed additionally to the neural network analyses to make the results for explainable, because explainability of AI can help to enhance trust of medical professionals in future AI systems [22,23].

In previous publications, we used publicly available data for ANNs. In this research, we used ANNs to predict the overall survival outcomes and molecular subtypes of a series of 106 cases from Tokai University Hospital, using gene expression data from the pancancer immune profiling panel, and validated the relevant marker with immunohistochemistry at protein level. We found that ANNs predicted survival and molecular subtypes with high accuracy.

## 2. Materials and Methods

### 2.1. Patients, Samples and Gene Expression Data

The series included 106 patients from Tokai University Hospital. This research complied with the Declaration of Helsinki and ethical principles regarding human experimentation. The Tokai University Institutional Review Board approved this research (protocol code IRB14R-080 and IRB-156).

**Figure 1:**
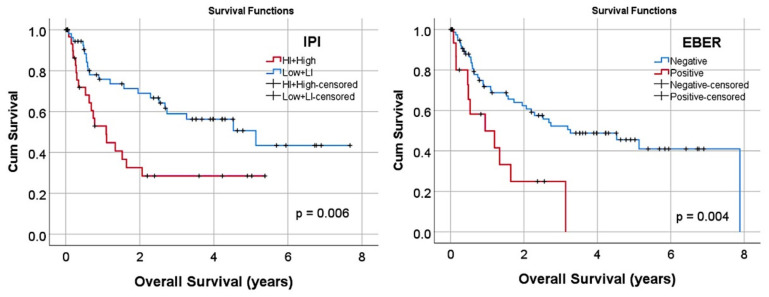
Overall survival according to the international prognostic index (IPI) and Epstein–Barr virus infection (EBER). Cases with IPI high–intermediate/high and EBER positivity were associated with poor overall survival (*p* < 0.01).

The cases were diagnosed following the criteria of the 2016 revision of the World Health Organization classification of lymphoid neoplasms [3] and corresponded to DLBCL morphology. The cases were selected from 2006 to 2016, being from the years 2008–2016 in 74% of the cases. This series of cases were from the rituximab-treatment era: they were mainly treated with R-CHOP (72.4%) or R-CHOP-like (22.4%) therapy. The main clinicopathological characteristics of the samples were as follows: male sex in 65/104 (62.5%); male/female ratio 65/39 (1.67); age range (23–97); age > 60 years in 70/104 (67.3%), and low International Prognostic Index (IPI) in 27/97 (27.8%), low–intermediate in 30/97 (30.9%), high–intermediate in 14/97 (14.4%), and high in 18/97 (18.6%). Based on the Lymph2Cx assay, the cell-of-origin subtypes were GCB in 51/104 (49%), ABC in 31/104 (29.8%), and unclassified in 22/104 (21.2%). Notably, in two cases, the assay result was nonassessable (total analyzed cases: 106). Epstein–Barr virus (EBV) positivity, assessed using EBV-encoded RNA (EBER) in situ hybridization (ISH), was negative in 79/98 (80.6%) and positive in 19/98 (19.4%) of the cases. The translocation status for *BCL2*, *MYC*, and *BCL6* was available in 72% of the cases: *BCL2* translocation positive (TL+) cases were 19/76 (25%), *MYC* TL+ were 19/76 (25%), and *BCL6* TL+ were 19/74 (25.7%). Cases with *MYC* and *BCL2* rearrangements, irrespective to *BCL6* translocation status, were 8/76 (10.5%). High-grade B-cell lymphoma with *MYC* and *BCL2* and/or *BCL6* rearrangements (HGBL) presented in 11 cases. The overall survival of this series according to the IPI and EBER is shown in Figure 1. As expected in a conventional series of DLBCL, high IPI and EBER-positive cases were associated with poor prognoses of patients.

Whole tissue sections from formalin-fixed paraffin-embedded tissue blocks, containing more than 70% tumoral cells, were outsourced to Celgene Corporation, where RNA extraction was performed and applied to the nCounter pancancer immune profiling panel (NanoString Technologies, Inc., Seattle, WA, USA). The molecular subtype was assessed using the Lymph2Cx gene expression panel (NanoString). This panel comprises 730 immune-oncology genes and 40 housekeeping genes. The list of housekeeping genes is shown in the Supplementary Materials.

In the Tokai series, immunohistochemistry using the Hans algorithm (CD10, BCL6, and MUM-1) was performed [7].

### 2.2. Artificial Neural Network Analysis

The multilayer perceptron analysis (MLP) used the normalized and log2 transformed gene expression data. We used the calibrated data, which had already been normalized to positive control, for the “housekeeping gene normalization” procedure. The calibrated data were the raw data multiplied by the calibration factors. The housekeeping gene normalization was calculated using the following formula: log2((normData(,i)/hkGeomMeans(i))). Notably, an alternative option was the following: log2((normData(,i)/hkGeomMeans(i))*scalingFactor). This scaling factor could be a constant, for instance, 1000.

**Figure 2:**
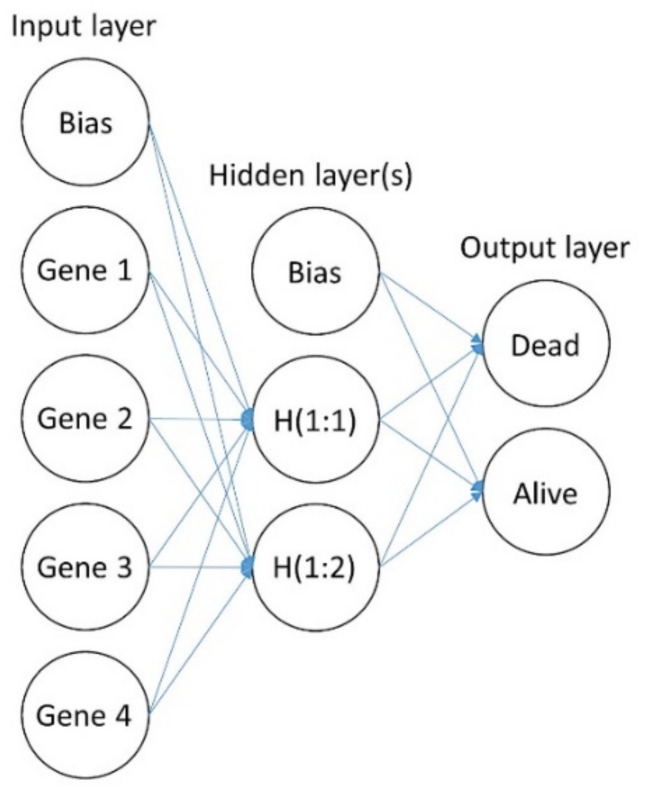
Basic structure of a multilayer perceptron (MLP). This figure shows the basic structure of an MLP artificial neural network, the same type of network used in this research. The network is characterized by a feedforward architecture with one hidden layer for predicting the overall survival outcome. The connections in the network flow forward from the input layer to the output layer without any feedback loop. The input layer contains the predictors (the gene expression data). The hidden layer contains unobservable nodes (units). The output layer contains the responses. The MLP network allows a second hidden layer. H (1:1) means hidden layer 1, node 1. In Table 1, the specific details of the neural networks are shown. For instance, the hidden layer of the MLP for overall survival had 6 nodes (H (1:1–6)), and that for the cell-of-origin molecular classification had 11 and 14 nodes.

**Figure 3:**
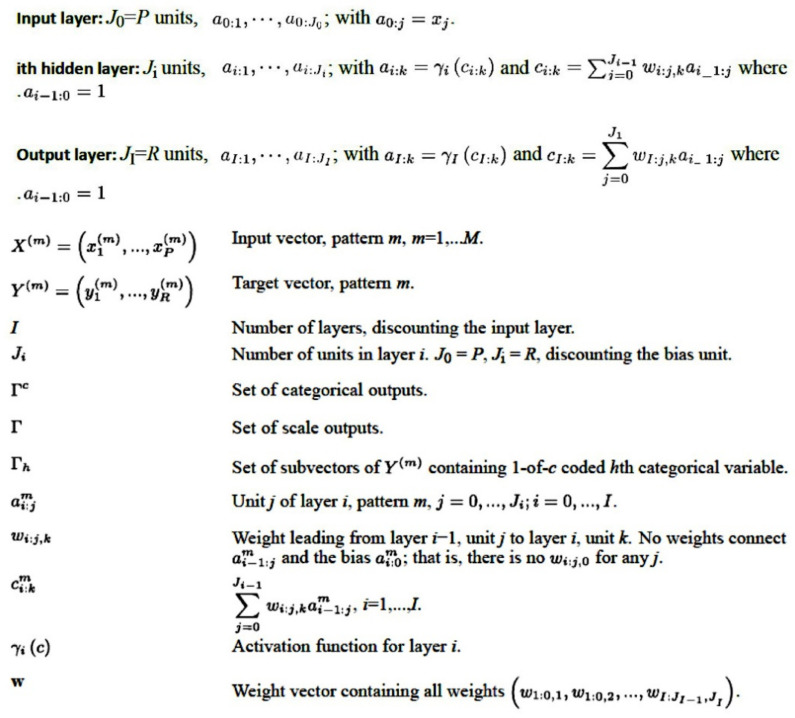
Architecture of the multilayer perceptron (MLP). The MLP is an artificial neural network that is characterized by a feedforward structure and supervised learning. The MLP network is a function of one or more predictors (known as inputs or independent variables) that minimizes the prediction error on one or more target variables (outputs). This figure shows the general architecture for the MLP network and the corresponding notation.

The complete procedure for MLP analysis was performed as we have previously described [10,11,12,13,19,20,21]. In the MLP procedure, predictive model for one or more dependent (target) variables is created based on the values of the predictor variables. The basic structure of an MLP is shown in Figure 2 and Figure 3 [10,11,12,13,19,20,21].

The dependent variables can be nominal, ordinal, or scale (continuous). The predictors can be specified as factors (categorical) or covariates (scale). In this study, the dependent variables were the overall survival outcome (dead vs. alive) and the Lymph2Cx molecular subtype (GCB, ABC, and Unspecified). The dependent variables were nominal, because their values represented categories with no intrinsic ranking. The predictors, which were the gene expression values of the pancancer immune profiling panel, were specified as covariates. The rescaling of covariates, which improves network training, was standardized. The database was partitioned by randomly assigning the cases based on relative numbers of cases: 70% to the training set and 30% to the testing set. In this analysis, the holdout partition was set at 0%. During the procedure, categorical predictors and dependent variables were temporarily recoded using one-of-*c* coding. When a variable has *c* categories, it is stored as c vectors: the first category (1,0,…,0), the next (0,1,0,…,0), and the final (0,0,…,0,1).

A series of parameters was set for architecture design. In the input layer, the nodes included the expression values of each gene. In the selection of the hidden layer, the number of layers (one or two), activation function (hyperbolic tangent or sigmoid), and number of units were specified. The hyperbolic tangent function had the form γ(*c*) = tanh(*c*) = (*e*^c^−*e*^−c^)/(*e*^c^ + *e*^−c^), and the sigmoid function, the form γ(*c*) = 1/(1 + *e*^−c^). The output layer contained the target (dependent) variables. The activation functions of the output layer were the identity (γ(*c*) = *c*), softmax (γ(*c*_k_) = exp(*c*_k_)/Σ_j_exp(*c*_j_)), the hyperbolic tangent, and the sigmoid. Notably, the activation function chosen for the output layer determined which rescaling methods were available. The rescaling of the dependent variables was standardized ((*x* − mean)/*s*), normalized ((*x* − min)/(max − min)), adjusted normalized ((2*(*x* − min)/(max − min)) − 1), and none.

The type of training determined how the network processed the records. The training types were batch, online, or minibatch. The batch, useful for small datasets, updated the synaptic weights only after passing all training data records. The online, more suitable for large datasets, updated the synaptic weights after every single training data records. The minibatch, best for medium-sized datasets, divided the training data records into groups of approximately equal size and updated the synaptic weights after passing one group. The synaptic weights were estimated using the optimization algorithms, the scaled conjugate gradient (only for batch training), and the gradient descent (for online, minibatch, and batch). The training options were different according to the type and the optimization algorithm. In the case of batch training and scaled conjugate gradient, the initial lambda was set at 0.0000005, the initial sigma at 0.00005, the interval center at 0, and the interval offset at ±0.5.

The network performance, which displays results used to determine whether the model is “good”, was assessed by the classification results, receiver operating characteristic (ROC) curve, cumulative gains chart, lift chart, predicted by observed chart, and residual by predicted chart.

The classification results, presented in Table 1, showed the classification table for each categorical dependent variable by partition and overall; the number of correctly and incorrectly classified cases were given.

The ROC curve is a graphical plot that shows the diagnostic ability of a binary classifier system as its discrimination threshold is varied. In a ROC curve, the true positive rate (sensitivity) is plotted as a function of the false positive rate (1–specificity). It is displayed for each categorical dependent variable. For each curve, the area under the curve (AUC) is also shown. The AUC is a measure of how well a parameter can distinguish between two diagnostic groups. A value of 0.5 means that the variable under study cannot distinguish between two groups. A perfect separation leads to an AUC of 1 [10,11,12,13,17,18,19,20,21,24].

For categorical dependent variables, the predicted-by-observed chart displays clustered boxplots of predicted pseudoprobabilities for the combined training and testing samples. The x axis corresponds to the observed response categories, and the legend to the predicted categories. Using 0.5 as the pseudoprobability cutoff for classification, the proportion of the boxplot above the 0.5 mark on the y axis represents correct predictions shown in the classification table. The proportion below the 0.5 mark represents incorrect predictions. When there are only two categories in the target variable, the first two boxplots are symmetrical about the horizontal line at 0.5 [10,11,12,13,19,20,21,25].

The cumulative gains chart shows the percentage of the overall number of cases in a given category “gained” by targeting a percentage of the total number of cases. The lift chart is derived from the cumulative gains chart; the values on the y axis correspond to the ratio of the cumulative gains for each curve to the baseline [10,11,12,13,19,20,21,25].

Using a sensitivity analysis, the independent variables were ranked according to their importance for predicting the dependent variable and in determining the neural network. The importance of an independent variable is a measure of how much the network’s model-predicted value changes for different values of the independent variable. Normalized importance is simply the importance values divided by the largest importance value and expressed as percentages [10,11,12,13,19,20,21,25].

The predicted value or category and the predicted pseudoprobability for each dependent variable were saved. The synaptic weights were exported to an xml file. The missing values were excluded from the analysis. As stopping rules, the maximum steps without a decrease in error were set at 1, the minimum relative change in training error was set at 0.0001, and the in-training error ratio was set at 0.001.

If it were necessary to exactly replicate the results, the same initialization value for random number generation, the same data order, the same variable order, and the same procedure settings should be used. Random number generation was used during the procedures of assignment of partitions, random subsampling for initialization of synaptic weights, random subsampling for automatic architecture selection, and the simulated annealing algorithm used in weight initialization and automatic architecture selection [10,11,12,13,19,20,21,25].

**Figure 4:**
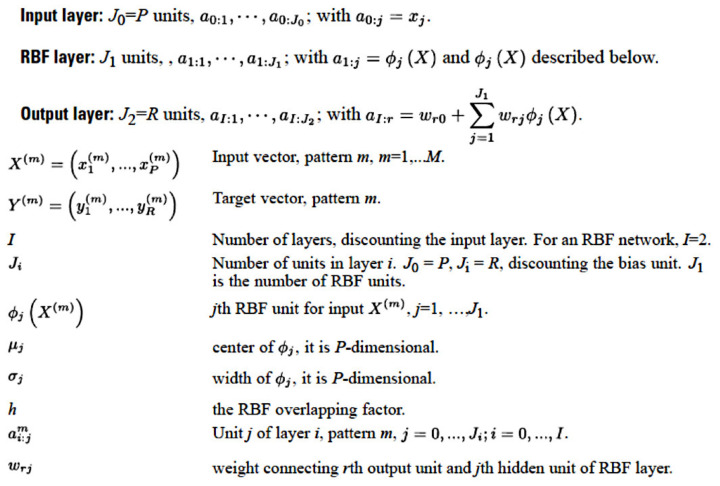
Architecture of the radial basis function (RBF). The RBF is a supervised feedforward learning network that is characterized by only one hidden layer. This figure shows the architecture of the three layers of the RBF network and the corresponding notation.

Radial basis function (RBF) analysis was also performed in a similar manner as MLP analysis. For the RBF analysis, the best number of units in the hidden layer was specified within a range or automatically computed, and the activation function was the normalized or the ordinary radial basis function. The overlap among hidden units was computed or specified. The user-missing values were excluded. The RBF algorithm is shown in Figure 4.

### 2.3. Statistical Analyses and Software

All statistical analyses were performed using several types of software, either for data processing, preanalysis, final analysis, or confirmation of results [10,11,12,13,17,18,19,20,21,24,25]:

- NSolver (version 4.0, NanoString, Seattle, Washington, USA); https://www.nanostring.com/products/analysis-solutions/ncounter-analysis-solutions/ (accessed on 29 November 2021);
- R (version 3.6.3) and R Studio (version 1.3.959, RStudio, Boston, MA, USA); https://www.rstudio.com/ (accessed on 29 November 2021);
- Excel (version 16, Microsoft, Redmond, WA, USA);
- EditPad Lite (version 8, Just Great Software Co. Ltd., Rawai Phuket, Thailand);
- JMP Statistical Discovery (version 14, SAS, Cary, NC, USA); https://www.jmp.com/ja_jp/home.html (accessed on 29 November 2021);
- IBM SPSS 26 and Modeler 18 (IBM, Armonk, NY, USA); https://www.ibm.com/jp-ja/products/spss-statistics (accessed on 29 November 2021).
- Gene Set Enrichment Analysis (GSEA) software (version 4.1.0, Broad Institute, UC San Diego, USA) [17,18]; https://www.gsea-msigdb.org/gsea/index.jsp (accessed on 29 November 2021); https://github.com/GSEA-MSigDB/gsea-desktop (accessed on 8 December 2021).
- Morpheus matrix visualization and analysis software (Broad Institute, Morpheus), https://software.broadinstitute.org/morpheus) (accessed on 29 November 2021);
- String (version 11, String consortium 2020) [19]; https://string-db.org/ (accessed on 29 November 2021).

Comparisons between groups were performed using crosstabulation (chi-square tests, including the Fisher’s exact test), and nonparametric tests for independent samples (Mann–Whitney, and Kruskal–Wallis H tests). Overall survival was calculated from the time of diagnosis to the time of death or the last alive follow-up time. Survival analysis was performed using the Kaplan–Meier and log rank tests, including the Breslow and Tarone–Ware tests. The hazard risks were calculated using Cox regression analysis. The association of the most relevant genes, which were highlighted in the neural network, with molecular subtypes was performed using binary logistic regression. Risk scores were calculated by multiplying the beta values of the multivariate Cox regression analysis for overall survival of each gene with the values of the corresponding gene expressions, as previously described [10,11,12,13,17,18,19,20,21,24,25]. These analyses were performed using mainly IBM SPSS. Survival analysis using R can be checked on the following web page: https://cran.r-project.org/web/views/Survival.html (accessed on 8 December 2021) [19]. Random forest is shown in http://genesrf.iib.uam.es/ and https://www.ligarto.org/rdiaz/software/software#varSelRF (based on R, accessed on 8 December 2021) [19]. All the analyses were performed on a Ryzen 7 3700X CPU workstation with 16 GB RAM and an NVIDIA GeForce GTX 1650 GPU.

**Figure 5:**
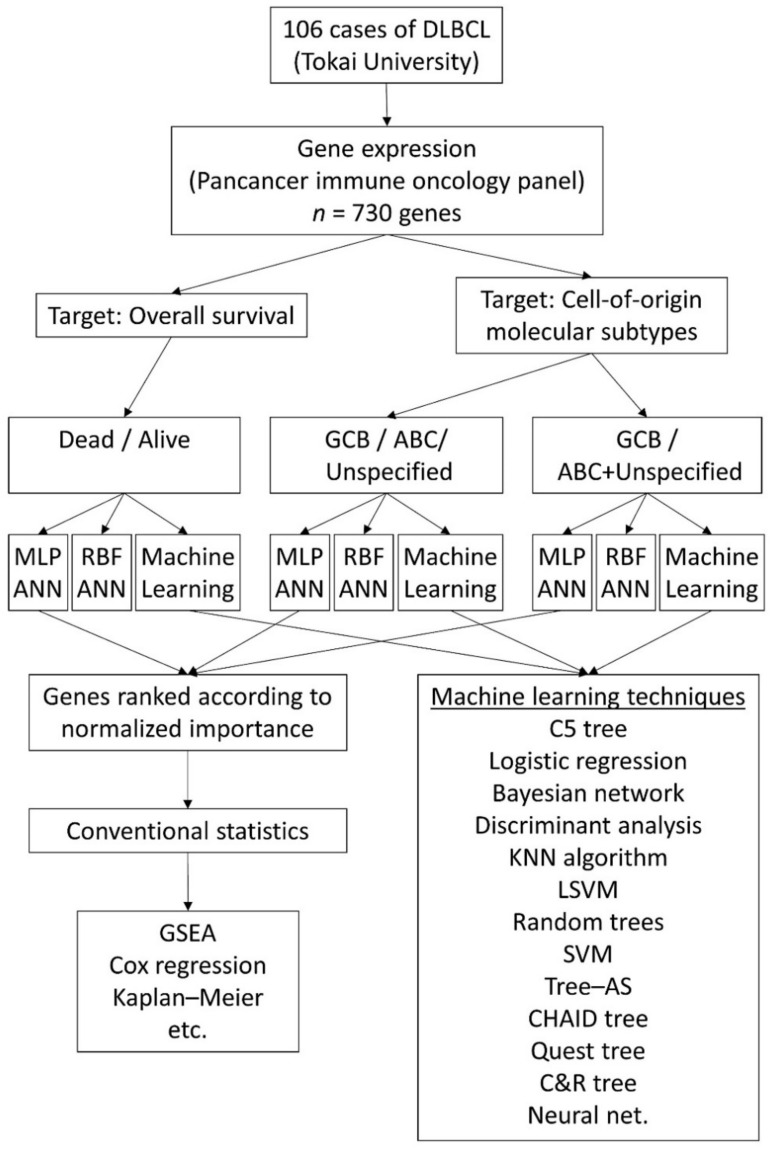
Data analysis workflow. This research used the gene expression data of 106 cases of DLBCL from Tokai University. The gene set was composed of 730 genes from the pancancer immune profiling panel. Two types of artificial neural network were used: multilayer perceptron (MLP) and radial basis function (RBF). Besides these, other machine learning techniques were included in the analysis in addition to conventional statistics.

The data analysis workflow is shown in Figure 5.

### 2.4. Immunohistochemistry

**Figure 6:**
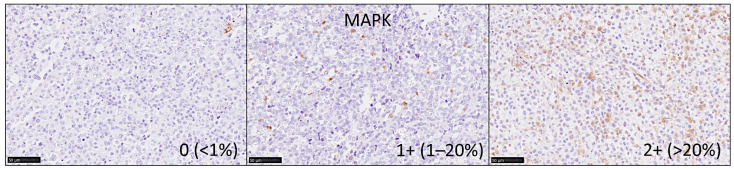
Immunohistochemistry for MAPK. The immunohistochemical signals were evaluated as an ordinal variable as 0 (no staining, <1%), 1 + (few scattered positive cells, 1–20%), or 2 + (abundant cells, >20%). Positive staining, brown color (DAB).

Immunohistochemistry for MAPK3 (ERK1) and MAPK1 (ERK2) was performed in a tissue microarray of 96 cases of DLBCL (Table A5). The clinicopathological characteristics of this series of 96 cases are shown in the Table A4. The microarray had been created from paraffin-embedded formalin-fixed tissue blocks and was stained using a rabbit monoclonal primary antibody against endogenous levels of phospho-p44/42 MAPK (Erk1/2) (Thr202/Tyr204) (#4370, Cell Signaling Technology K.K. Tokyo, Japan). The staining was performed using a Leica Bon-Max slide stainer (Leica Biosystems K.K. Tokyo, Japan) following the manufacturer’s instructions [26], Bond epitope retrieval solution 1 (pH 6.0, 30 min., #AR9961, Leica), and at a 1:400 dilution. After staining, the slides were scanned in a NanoZoomer S360 digital slide scanner (#C13220-01, Hamamatsu K.K. Hamamatsu, Japan) and visualized using the NDP.view2 viewing software (#U12388-01, Hamamatsu). The MAPK-positive cells had morphology compatible with macrophages or dendritic cells. The immunohistochemical signals were evaluated as an ordinal variable as 0 (no staining, <1%), 1 + (few scattered positive cells, 1–20%), or 2 + (abundant cells, >20%) (Figure 6). The correlation of MAPK as an ordinal variable with the molecular subtype according to the Hans classifier was performed using binary logistic regression. Additional markers for correlation with MAPK were LMO2 (mouse monoclonal, 299B, CNIO, Spain), CD163 (10D6, Leica), CSF1R (FER216, CNIO, Spain), and PD-L1 (E1J2J, Cell Signaling) [12,16].

## 3. Results

### 3.1. Prediction of the Overall Survival Outcome (Dead/Alive)

#### 3.1.1. Analysis Using the 730 Genes of the Pancancer Panel

The 730 genes of the pancancer immune profiling panel were used to predict the overall survival outcome using a multilayer perceptron (MLP) analysis. Table 1 shows the detailed information of the artificial neural network, including case processing; characteristics of the input, hidden, and output layers; a model summary for training and testing; classification; and the area under the curve. The training set included 72 of 105 cases (67%) and the testing set included 33 of 105 cases (31%). The performance of the network was satisfactory, with only 15.3% incorrect predictions. The percentages of correct classifications in the training and validation sets were 84.75% and 81.8%, respectively. The area under the curve was 0.898 for both alive and death survival outcome. According to the normalized importance, the top 10 most relevant genes for this model were *CD55, ARG1, SPANXB1, CTAG1B, IFNA8, CASP1, IL2, TNFSF12, ANP32B*, and *CTSG* (Table 2). Among the following genes on the list, 11–20, other relevant genes in the pathogenesis of cancer were identified, such as *REL* and *CD8A*.

#### 3.1.2. Analysis Using the Top 20 Genes of the MLP

**Figure 7:**
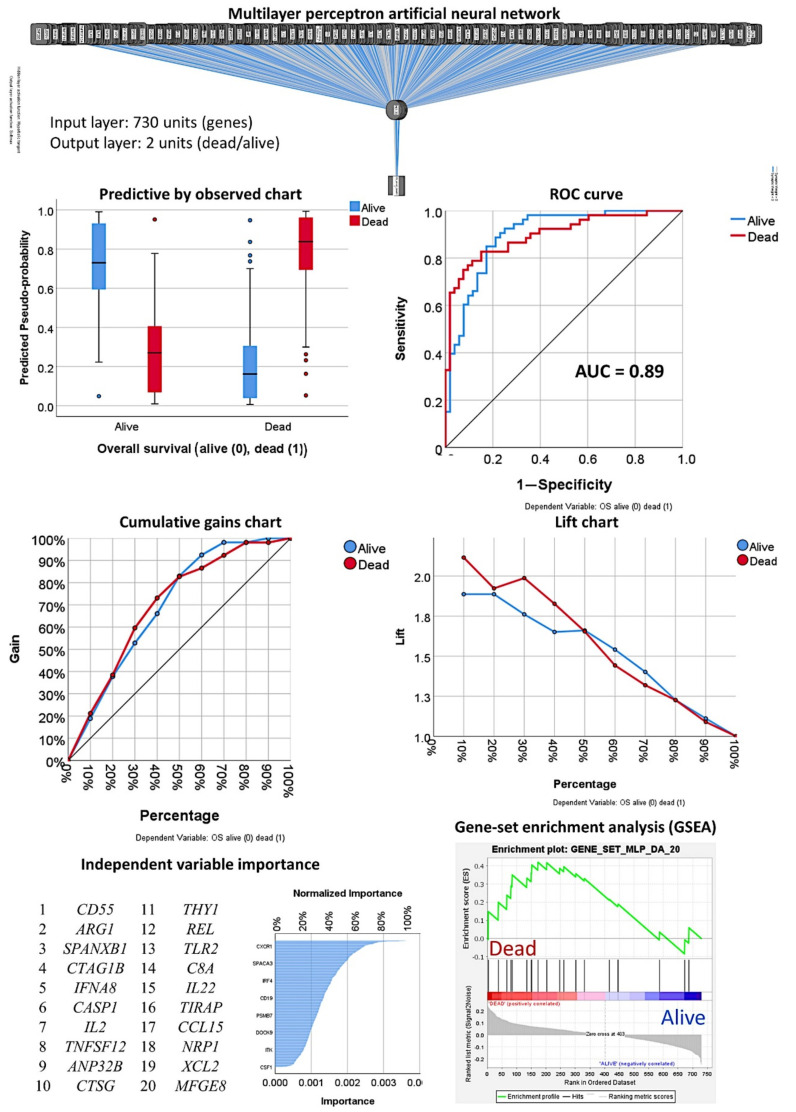
Multilayer perceptron artificial neural network for predicting overall survival. The neural network predicted the overall survival outcome as dead/alive using the 730 genes of the pancancer immune oncology profiling panel. The network performance can be checked using several parameters such as the area under the curve (AUC), which had a value of 0.89. The network performance outputs are the predictive by observed chart, the cumulative gains chart, and the lift chard. The genes were ranked according to their normalized importance for prediction of the overall survival outcome, as shown in the independent variable importance chart. The top 20 genes are listed. GSEA showed enrichment toward the dead phenotype for some genes.

**Figure 8:**
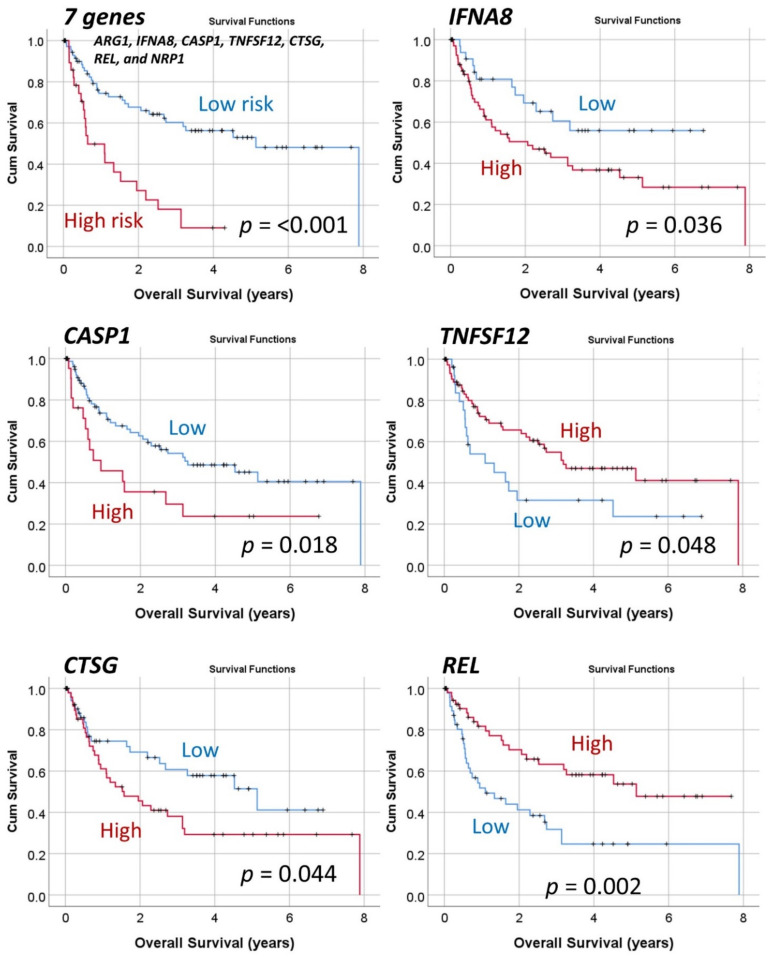
Overall survival according to the top genes of the MLP analysis. Because of the MLP and multivariate Cox regression analysis a final set of 7 genes were highlighted. Using a risk-score formula, the cases were divided into high and low-risk groups that had different overall survival (*p* < 0.001). Additionally, using a cutoff for the gene expression values, overall survival plots were calculated for each highlighted gene.

To comprehend and trust the results of the output created by the neural network, a concept known as explainable artificial intelligence (XAI), the top 20 genes identified by the MLP were correlated with the overall survival of patients. The correlation used univariate (Table A1) and multivariate Cox regression analyses (Table 3) and gene-set enrichment analysis (GSEA). The GSEA showed enrichment toward the dead phenotype, confirming that some of the genes associated toward patients who died (Figure 7). In the multivariate regression analysis, seven genes were the most relevant: *ARG1, IFNA8, CASP1, TNFSF12, CTSG, REL*, and *NRP1* (Table 3, Step 14 (last)). The overall survival plot for each gene is shown in Figure 8. Finally, using a risk-score formula with the gene expression of 20 genes or the 7 genes, two risk groups could be defined with different overall survival outcomes (Figure 3). The high-risk group was characterized by higher expression of *CD163*, which is a marker of M2-like tumor-associated macrophages (TAMs), and *MYD88*, which is a marker of NF-kappa-B activation, cytokine secretion, and inflammatory response. High-risk vs. low-risk group, 1.7 ± 3.5 vs. 0.4 ± 1.7 (*p* = 0.002) and 1.2 0.7 vs. 0.9 0.4 (*p* = 0.008). Table A3 shows the immune oncology annotations of the top 20 genes.

**Figure 9:**
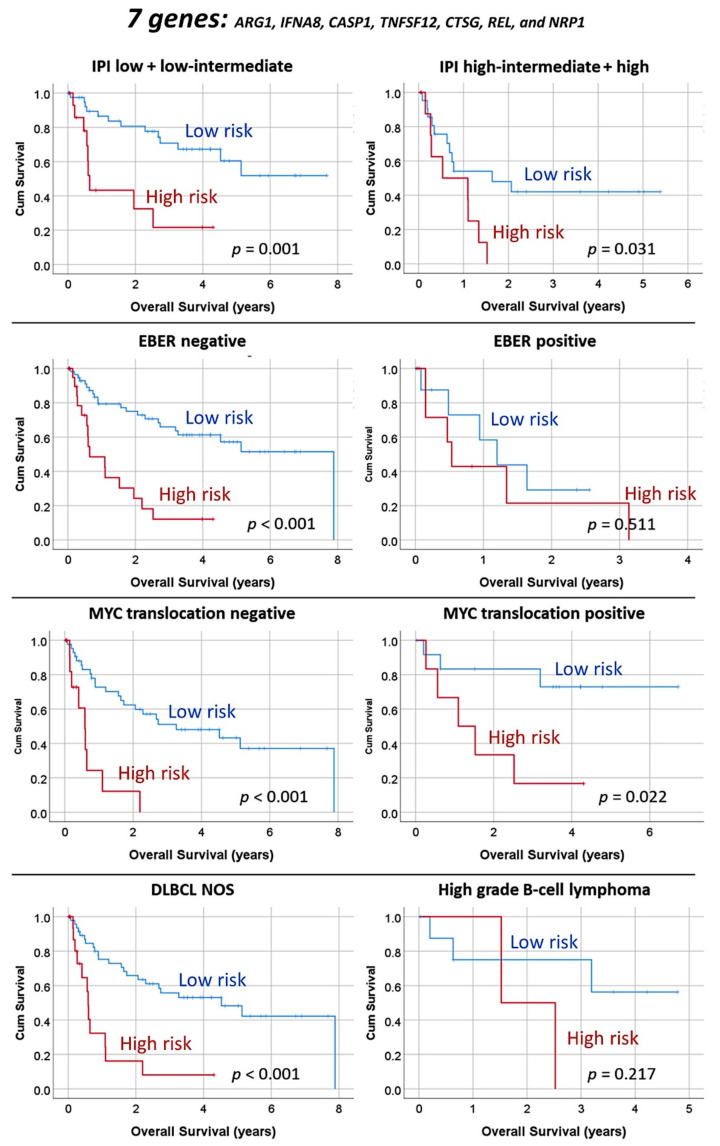
Overall survival according to the top genes of the MLP analysis in DLBCL subtypes/entities. As a result of the MLP and the multivariate Cox regression analysis a final set of 7 genes were highlighted. Using a risk-score formula, the cases were divided into high and low-risk groups that had different overall survival when stratifying for IPI, Epstein Barr virus infection (EBER), *MYC* rearrangement and High-grade B-cell lymphoma with *MYC* and *BCL2* and/or *BCL6* rearrangement.

The predictive value for overall survival of these seven genes was evaluated in the different subtypes/entities of DLBCL using the same risk groups and cutoffs (Figure 9). The predictive value was kept in within the IPI L+LI and H+HI strata, within the EBER-negative cases (but not in the EBER-positive cases), within *MYC* translocation positive and negative cases, and within the non-High-grade B-cell lymphoma with *MYC* and *BCL2* and/or *BCL6* rearrangements (i.e., DLBCL NOS) (but not in the 11 High-grade B-cell lymphomas).

#### 3.1.3. Multivariate Analysis Using the Set of Seven Genes and Clinicopathological Variables

A multivariate Cox regression analysis for prediction of the overall survival using the variables of the final set of seven genes, IPI, and EBV was calculated. The results were as follows: set of seven genes, *p* < 0.001, hazard risk (HR) = 3.6 (95% CI = 1.8–7.1); IPI, *p* = 0.055, HR = 1.9 (0.9–3.6); and EBER, *p* = 0.054, HR = 0.054 (0.9–4.9).

When the molecular subtypes (GCB vs. ABC+Unspecified) were included in the equation, the results were as follows: set of seven genes, *p* < 0.001, HR = 2.3 (1.1–5.2); EBER, *p* = 0.036, HR = 2.3 (1.1–5.2); IPI, *p* = 0.134, HR = 1.7 (0.9–3.2); and molecular subtypes, *p* = 0.107, HR = 1.8 (0.9–3.4).

Finally, when the set of seven genes was included in the equation with the IPI, EBV, molecular subtypes, and High-grade B-cell lymphoma, only the set of seven genes (*p* < 0.001, HR = 5.4) and EBER (*p* = 0.006, HR = 5.3) retained prognostic relevance. Therefore, the final set of seven genes was an independent prognostic factor.

In this series, both the IPI and EBER had prognostic relevance (Figure 1). Within the variables that make up the IPI (age >60, Ann Arbor stage III–IV, ECOG performance status ≥2, serum LDH level >1 x normal, and > 1 extranodal site), a univariate Cox regression analysis revealed that for overall survival, only the stage (*p* = 0.039, HR = 2.0), LDH (*p* = 0.024, HR = 2.4), and >1 extranodal site (*p* = 0.007, HR = 3.1) had prognostic relevance. Since these variables were within the IPI, in the final Cox model, they were not included.

**Figure 10:**
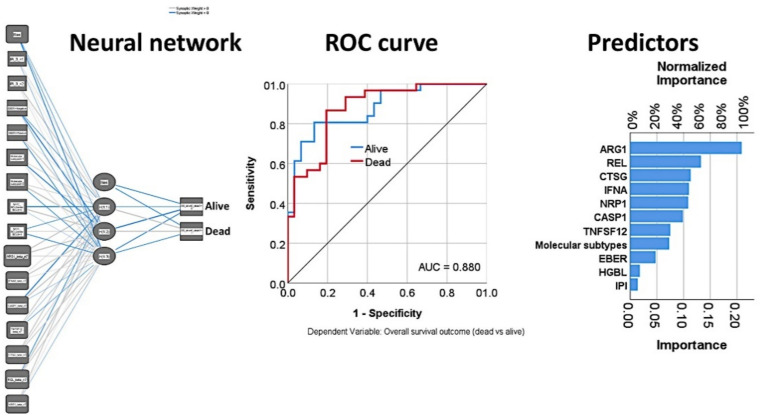
Multivariate overall survival analyses. The set of 7 genes was used in addition to the IPI, EBER, molecular subtypes, and HGBL to predict the overall survival outcome (dead/alive). A multilayer perceptron analysis successfully classified the cases based on those parameters, with a network performance having an area under the curve (AUC) of 0.880. The variables were ranked according to their normalized importance for predicting the prognosis. The most relevant predictors were *ARG1*, *REL*, and *CTSG*.

Notably, a multilayer perceptron analysis could be used to perform the multivariate analysis in a nonlinear manner (Figure 10). The input variables (predictors, 15 units) were the IPI, EBER, molecular subtypes, High-grade B-cell lymphoma, and the seven genes of the set. The output variable was the overall survival outcome (dead vs. alive, two units). The hidden layer had one layer with three units. The activation function was the hyperbolic tangent in the hidden layer and softmax in the output layer. The network performance was good, with an ROC area under the curve of 0.880 and 84.4% correct classification. The most important factors, according to their normalized importance (NI), for predicting the overall survival outcome (dead/alive) were as follows: *ARG1* (100% NI), *REL* (63.5%), *CTSG* (54.2%), *IFNA8* (52.7%), *NRP1* (52.0%), *CASP1* (47.3%), *TNFSF12* (36.0%), molecular subtypes (34.9%), EBER (22.8%), high-grade B-cell lymphoma (8.7%), and IPI (6.7%).

#### 3.1.4. Additional Machine Learning Analyses

**Figure 11:**
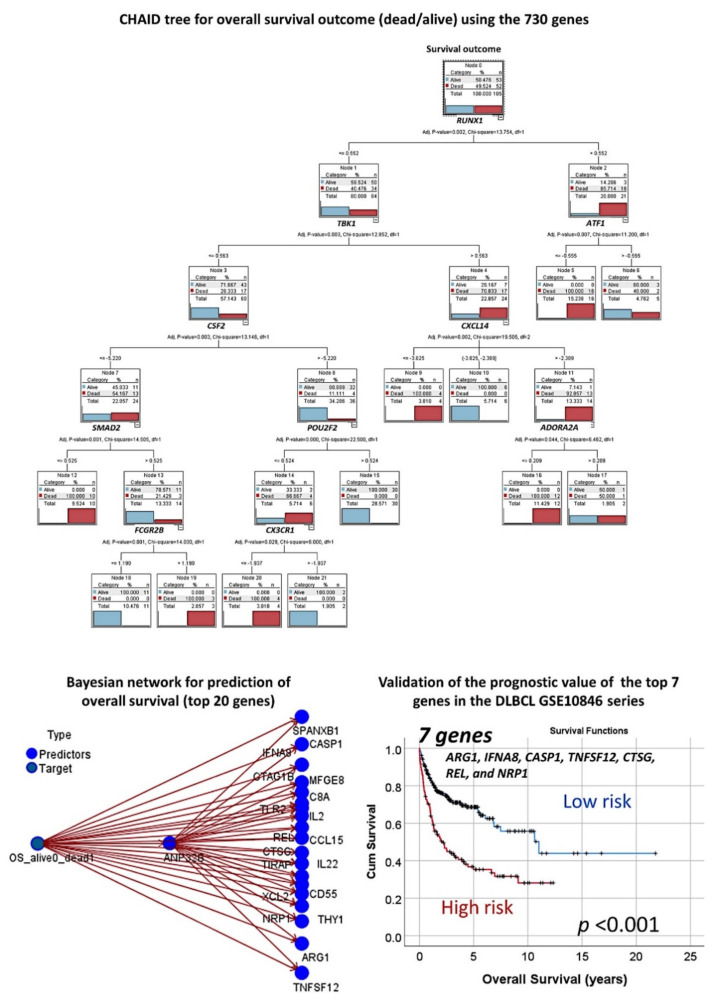
Other machine learning techniques for predicting overall survival. In addition to the artificial neural networks, other machine learning techniques were used. This figure shows the results of the CHAID decision tree and the Bayesian network. CHAID, or chi-squared automatic interaction detection, is a classification method for building decision trees by using chi-squared statistics to identify optimal splits. A Bayesian network is a graphical model that displays variables (nodes) in a dataset and the probabilistic, or conditional, independencies between them. Causal relationships between nodes may be represented but the links (arcs) do not necessarily represent direct cause and effect. Finally, the predictive value of the final set of 7 genes was tested in an independent series of DLBCL of 414 cases, and the results were reproducible.

In addition to artificial neural networks, other machine learning techniques were used. Table 4 shows the overall accuracy of the tests and the numbers of fields that were used in the final model. Logistic regression, discriminant analysis, and SVM predicted the overall survival outcome with overall accuracies of 100% using the 730 genes of the panel. Besides these, decision trees also predicted overall survival with high accuracy, and above 95% in the case of CHAID and C5 trees. The CHAID method had the best accuracy among the decision trees and used only 10 genes in the model, which were *RUNX1, TBK1, ATF1, CSF2, CXCL14, SMAD2, POU2F2, ADORA2A, FCGR2B*, and *CXCR1* (Figure 11).

The modeling for overall survival using other machine learning techniques was repeated using only the top 20 genes identified from the multilayer perceptron analysis. The most accurate model was the Bayesian network, which had an overall accuracy of 93% (Figure 11), followed by the C&R tree (77%), C5 tree (70%), KNN algorithm (70%), and logistic regression (68%).

#### 3.1.5. Validation in an Independent Series of DLBCL

Validation of the prognostic value of the set of genes identified in the Tokai series was performed using the GSE10848 series, which includes 414 cases. Using the risk-score formula [20] with the gene expression of the 20 genes or the 7 genes, two risk groups were defined, which had different overall survival outcomes (log rank *p* < 0.0001, HR = 3.6 and *p* < 0.0001, HR = 2.4, respectively) (Figure 11).

### 3.2. Prediction of the Three Molecular Subtypes (GCB, ABC, and Unspecified)

**Figure 12:**
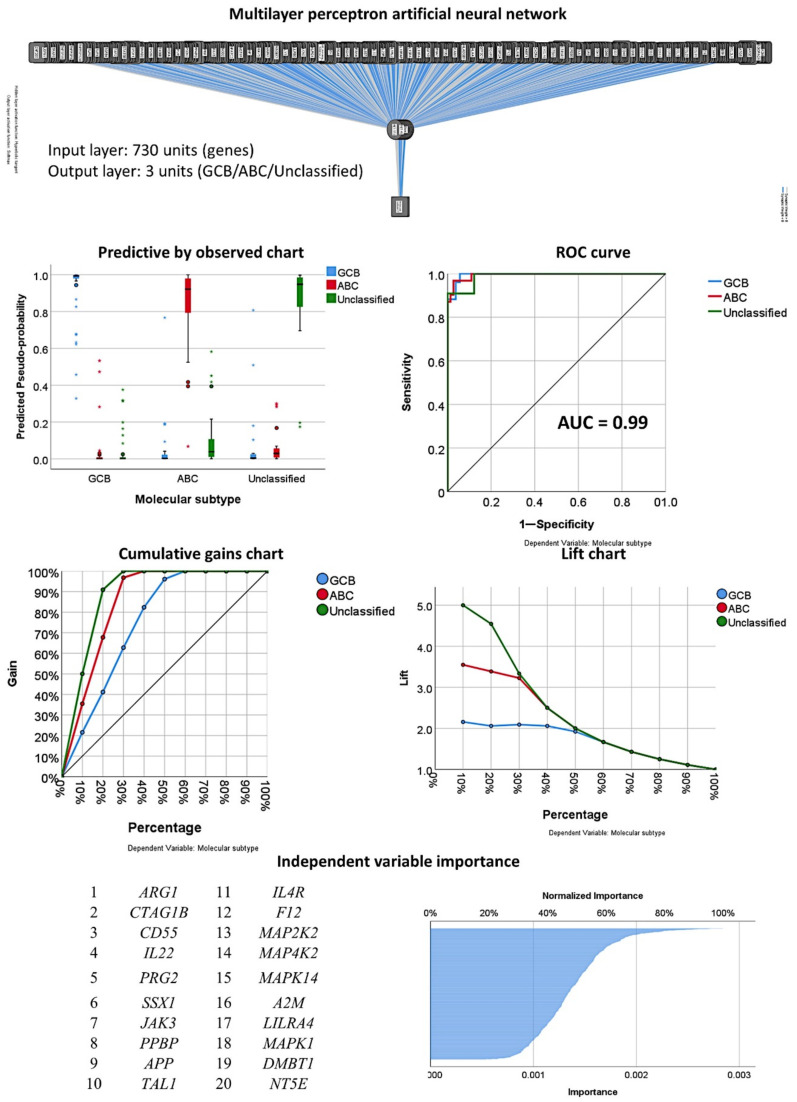
Multilayer perceptron analysis for predicting molecular subtypes (GCB, ABC, unspecified). The neural network predicted molecular subtypes as GCB, ABC, and Unspecified using the 730 genes of the pancancer immune oncology profiling panel. The network performance was checked using several parameters, such as the area under the curve (AUC), which had a value of 0.99. The genes were ranked according to their normalized importance for prediction, as shown in the independent variable importance chart. The top 20 genes are listed. The molecular subtypes were based on the Lymph2Cx assay.

The multilayer perceptron analysis correlated the 730 pancancer immune profiling genes with the molecular subtypes of GCB, ABC, and Unspecified. The artificial neural network successfully predicted the molecular subtypes with high accuracy (Figure 12). The classification was correct in 98.7% of the cases in the training set and 81.5% of those in the testing set. The area under the curve was 0.99 for both GCB and ABC and 0.98 for the Unspecified group. Table 1 shows the details of this artificial neural network. According to their normalized importance for predicting the molecular subtype, the top most relevant genes were *A2M, ABCB1, ABL1, ADA, ADORA2A, AICDA, AIRE, AKT3, ALCAM*, and *AMBP* (Table 2).

Several machine learning techniques were applied, and logistic regression, discriminant analysis, and SVM predicted the molecular subtypes with overall accuracies of 100% using the 730 genes of the panel. Decision trees also managed to predict the molecular subtypes. The C5 and CHAID trees used 7 and 8 genes, respectively, with overall accuracies of 96% (Table 4).

### 3.3. Prediction of the Two Molecular Subtypes (GCB, ABC+Unspecified)

#### 3.3.1. Analysis Using the 730 Genes of the Pancancer Panel

**Figure 13:**
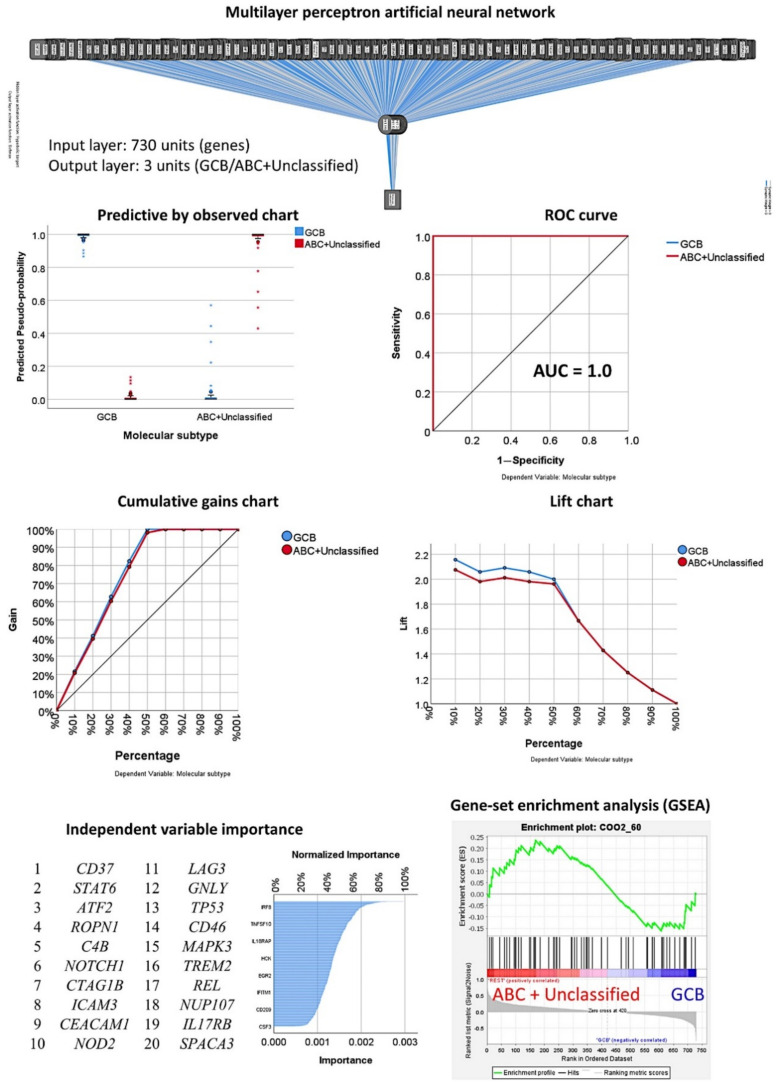
Multilayer perceptron analysis for predicting molecular subtypes (GCB vs. ABC+Unspecified). The neural network predicted the molecular subtypes as GCB and ABC+Unspecified using the 730 genes of the pancancer immune oncology profiling panel. The network performance was checked using several parameters, such as the area under the curve (AUC), which had a value of 1.0. The genes were ranked according to their normalized importance for prediction, as shown in the independent variable importance chart. The top 20 genes are listed. GSEA analysis had a sinusoidal-like shape, with some genes associated with the GCB and others with the ABC+Unspecified phenotype.

**Figure 14:**
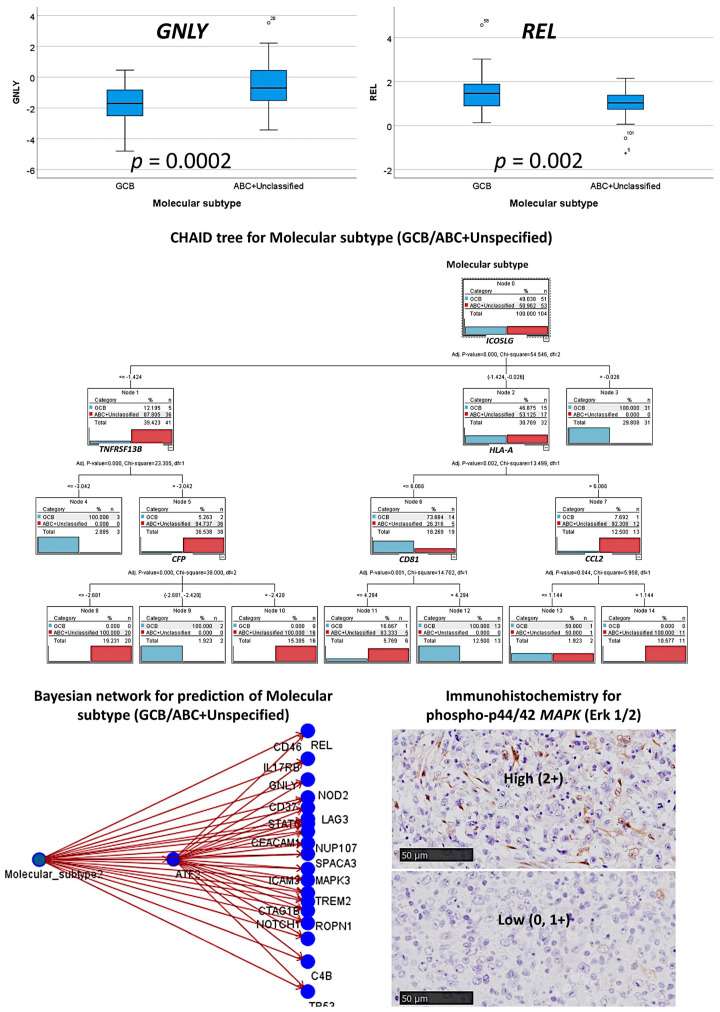
Other machine learning techniques for predicting molecular subtypes. The different gene expression levels between the two molecular subtypes are shown in a boxplot figure. In addition to artificial neural networks, other machine learning techniques were used. This figure shows the results of the CHAID decision tree and the Bayesian network. Finally, the predictive value of the final set of MAPK was tested in an independent series of DLBCL of 96 cases from Tokai University, and the results confirmed the association with the GCB phenotype. The expression of MAP3K was correlated with LMO2 and M2-like tumor-associated macrophage markers including CSF1R, CD163, and PD-L1. MAP3K correlated with LMO2 (odds ratio = 2.8, *p* = 0.039). Interestingly, though MAP3K showed histological expression similar to that of macrophages, no correlation was found with the markers (CD163, CSF1R, TNFAIP8, CASP8, PD-L1, PTX3, and IL-10).

The multilayer perceptron analysis correlated the 730 pancancer immune profiling genes with the molecular subtypes as GCB versus ABC+Unspecified (Table 1, Figure 13 and Figure 14). In comparison to the other analyses, this artificial neural network had the best prediction accuracy, with lower percentages of incorrect predictions and cross-entropy errors. The percentages of correct classification were 100% for the training set and 96.4% for the testing set. The areas under the curve were 1.0 for both GCB and ABC+Unspecified. Table 2 shows the most relevant predictive genes identified by the artificial neural network. The top 10 genes were *CD37, STAT6, ATF2, ROPN1, C4B, NOTCH1, CTAG1B, ICAM3, CEACAM1*, and *NOD2*. Other relevant genes present within the 11-20 top genes were *LAG3, TP53, MAPK3*, and *REL*.

MAP3K was tested at the protein level using immunohistochemistry in another series; the clinicopathological characteristics of this series are shown in Table A5. The frequencies with immunohistochemistry for phospho-p44/42 MAPK (Erk1/2) (Thr202/Tyr204) were as follows: 0, 32/90 (35.6%); 1+, 30/90 (33.3%); and 2+, 28/90 (31.1%). The immunohistochemistry confirmed the relevance of this marker as highlighted in the neural network. High expression of MAP3K associated with a GCB phenotype (odds ratio of non-GCB = 0.543, 95% CI 0.3–0.96, *p* = 0.037). The expression of MAP3K was correlated with LMO2 and macrophage markers including CSF1R, CD163, TNFAIP8, CASP8, PTX3, and PD-L1. MAP3K correlated with LMO2 (a marker of the germinal center) (odds ratio = 2.8, 95% CI: 1.1–7.2, *p* = 0.039). Interestingly, though MAP3K showed histological expression similar to that of macrophages, no correlation was found with markers of M2-like tumor-associated macrophages (all *p* > 0.05).

#### 3.3.2. Analysis Using the Top 20 Genes of the MLP

In Table 5, the associations between the top 20 genes and the molecular subtypes, as calculated using multivariate binary logistic regression, are shown. In the final model, the most relevant genes positively associated with the ABC+Unspecified subtype were *CD37*, *GNLY*, and *IL17RB*; *STAT6* and *REL* were inversely correlated. Table A2 shows the univariate analysis results. Table A4 shows the immune oncology annotations. The GSEA analysis showed a sinusoidal-like shape, with some markers associated with ABC+Unspecified and others with GCB (Figure 5).

#### 3.3.3. Additional Machine Learning Analyses

Other machine learning techniques also predicted the molecular subtype with high accuracy. Some included the 730 genes in the model, such as logistic regression, discriminant analysis, SVM, and KNN algorithm. However, the CHAID and C5 trees used six and five genes, respectively (Table 5, Figure 13).

The modeling for overall survival using other machine learning techniques was repeated using only the top 20 genes identified by the multilayer perceptron analysis. The most accurate model was the Bayesian network, which had an overall accuracy of 93% (Figure 14), followed by the C5 tree (88%), logistic regression (68%), and discriminant analysis (86%).

### 3.4. Artificial Neural Network Analysis Using the Radial Basis Function

All of the data were reanalyzed with a radial basis function (RBF) ANN as in the multilayer perceptron analysis. The neural network predicted both the overall survival outcome and the molecular subtypes. The network performance for the survival outcome was poor (AUC of 0.628). However, the performances for the molecular subtypes were acceptable (0.83 and 0.85). Since the performance of the multilayer perceptron was better, the results for the RBF are not shown in this manuscript.

## 4. Discussion

DLBCL is heterogeneous in terms of morphological features, genetic alterations, biological characteristics, and prognosis [1,2,3]. The preferred treatment is chemoimmunotherapy with R-CHOP (rituximab, cyclophosphamide, doxorubicin, vincristine, and prednisone). Gene expression has been extensively studied in DLBCL using microarray technology [27]. The cell of origin classification, which is based on unsupervised clustering, has dominated the field. Despite the recent advances in diagnosis and treatment, there is a need to find prognostic markers.

This research analyzed the microarray data using a novel approach based on artificial neural networks and included conventional strategies to make the results more explainable. Originally, the molecular subtypes were defined using frozen tissue. Using the Lymph2Cx assay [28], the classification can now be conducted using formalin-fixed paraffin-embedded tissue biopsies [9,29]. The Lymph2Cx panel included 37 genes, 32 “endogenous” and 5 “controls”. Among the “endogenous”, markers of the conventional Hans classifier are present, such as *MME* (CD10), *BCL6*, and *IRF4* (MUM-1). Other interesting markers for the pathology of DLBCL are *BTK, MYC, CARD11, LMO2, TP53,* and *MYD88*. This research classified the cases based on the Lymph2Cx assay: the frequency of the GCB subtype was 51/104 (49%), ABC 31/104 (29.8%), and Unspecified 22/104 (21.2%).

This research used a pancancer immune profiling panel, which was fully compatible with clinically relevant formalin-fixed, paraffin-embedded (FFPE) tumor sections. This panel had an immune cell coverage that included B-cells, T-cells, CD4-positive Th1 cells, regulatory T-lymphocytes (Tregs), CD8-positive cytotoxic T-lymphocytes, exhausted CD8-positve T-lymphocytes, cytotoxic cells, dendritic cells, macrophages, mast cells, neutrophils, and NK cells. B-cells are primary mediators of the humoral immune response. T-cells mediate cell-based immunity using cytokines and directly kill target cells. CD4-positive Th1 cells release IL2 and interferon gamma and stimulate CD8-positive cytotoxic T-lymphocytes, NK cells, and macrophages. Tregs play an important role in suppressing immune responses, affecting both B- and T-cells [14]. Using the genes of this panel, we predicted the overall survival outcome and molecular subtypes with high accuracy, and the top 20 genes influencing each prediction were highlighted. The annotation of these genes regarding the immune profiling panel is shown in the Appendix A. For example, the top 20 genes that predicted overall survival belonged to the immune response, CT antigen and cell type specific (Th and mast cells). Within the immune response, the most relevant categories were cell functions (*IL2, ANP32B/ARPRIL*, and *NRP1*), chemokines (*TNFSF12, CCL15,* and *XCL2*), and regulation (*IL2, CTSG*, and *TIRAP*). Regarding the molecular subtypes, the annotations were immune response, cell type Th and cytotoxic cells, and CT antigens. The most relevant immune response categories were regulation (*STAT6, NOTCH1, ICAM3, LAG3*, and *REL*) and T- and B-cell functions (*STAT6, LAG3,* and *TP53*). Therefore, we showed that the immune response is important for survival and molecular subtype classification in the pathogenesis of DLBCL.

Artificial neural networks are the chosen tool for many predictive data mining applications because they are easy to use, flexible, and powerful [17,18]. Predictive neural networks are especially useful when the underlying processes are complex, such as the pathological background of DLBCL. This research used two types of neural networks, the multilayer perceptron (MLP) and radial basis function (RBF). The type of data and the level of complexity define the procedure to use. While the MLP procedure can find more complex relationships, RBF is faster [17,18]. This research used both types, but we found that the MLP made more accurate predictions. Therefore, the analysis was based mainly on the MLP results.

Artificial neural networks are used in predictive applications and are supervised in the sense that the model-predicted results can be compared against known values of the target variables [17,18]. An advantage of neural networks is that they make minimal demands on the model structure and assumptions, unlike traditional statistical methods. The traditional linear regression model, when using the least-square method and storing the regression coefficients, is a special case of certain neural network. However, it has a rigid model structure and a set of assumptions that are imposed before learning from the data. However, neural networks are flexible. The tradeoff is that the synaptic weights are not easily interpretable [17,18]. For example, the synaptic weights for the most relevant gene for predicting the overall survival outcome, *CD55*, were as follows: -0.441 for H(1:1), -0.204 for H(1:2), 0.168 for H(1:3), 0.199 for H(1:4), -0.458 for H(1:5), and -0.733 for H(1:6). The synaptic weight informs about the amplitude or the strength of the connection between two nodes (neurons). Since ANNs are black-box models because of their multilayer nonlinear structure, the explanation of the underlying process that produces the relationship between the dependent (target) and independent (predictors) variables is unintelligible, nontransparent, and untraceable by humans [30]. The overall survival outcome and the molecular subtypes of patients with diffuse large B-cell lymphoma (DLBCL) were predicted with high accuracy, and the most relevant genes were highlighted using nonlinear analysis. To make the results more understandable, i.e., explainable artificial intelligence (XAI), several machine learning methods were applied. A thorough evaluation of the relationships between the predictors and the predicted variables in these methods explained the underlying process of the neural network. For example, the MLP highlighted 20 genes with high capability to predict the overall survival outcome, and using conventional analyses such as GSEA, we confirmed the association with bad prognosis. Multivariate Cox regression analysis reduced the list to seven genes, with *ARG1, TNFSF12, REL*, and *NRP1* associated with good (HR < 1) and *IFNA8, CASP1*, and *CTSG* with bad prognosis (HR > 1). As individual markers, these genes also predicted the prognosis, as shown in the Kaplan–Meier plots. Additionally, the risk-score formula integrated all genes, and two groups with different risk could be found among the results. Macrophages release interferon alpha-8 (*IFNA8*) [26], and we found that the high-risk group was associated with high expression of *CD163*, which is a marker of M2-like tumor-associated macrophages (TAMs). Caspase-1 (*CASP1*) is involved in various inflammatory processes and initiates programmed cell death [31], and we found that the high-risk group also associated with high *MYD88* expression. Cathepsin G (*CTSG*) belongs to the complement pathway [32]. It has been related to oral squamous cell carcinoma [33], is broadly expressed in acute myeloid leukemia, and is an effective immunotherapeutic target [34]. Finally, in this research the pancancer immune profiling panel predicted with high accuracy molecular subtypes, and the most relevant markers for the ABC/non-GCB phenotype were *CD37, GNLY*, and *CD46*. Membrane cofactor protein (*CD46*) acts as a costimulatory factor for T-cells, which induces the differentiation of Tregs [32]. Therefore, the data showed that the immune microenvironment plays an important role in GCB and non-GCB differentiation, as shown in the germinal center dynamics under physiological conditions.

We recently described that high expression of PTX3 was associated with poor prognosis in DLBCL [25]. Though the immunohistochemistry of MAPK showed a macrophage-like pattern, no correlation was found between MAPK and PTX3. Similar results were found for the TNFAIP8 marker [12]. We also previously described the gene expression of High-grade B-cell lymphoma [35]. In this research, we identified seven genes that predicted the overall survival of patients of non-High-grade B-cell lymphoma cases. Therefore, our data suggested that AID is a poor prognostic marker of High-grade B-cell lymphoma with *MYC* and *BCL2* and/or *BCL6* rearrangements, as it has a different pathological background.

Applying artificial intelligence for the analysis of gene expression not only is useful in the analysis of individual entities, but allows differentiating between different lymphoma subtypes, as we showed in non-Hodgkin lymphomas [19]. Deep neural networks are characterized by having a multilayer nonlinear structure (i.e., black-box model). Therefore, neural networks are criticized as being nontransparent because their predictions are not traceable by humans. In this research, we combined artificial neural networks and machine learning to make the results more understandable (explainable [22]). In the future, explainable artificial intelligence (XAI) may enable human users to understand, and hence trust, artificial intelligence methods and results of high prediction accuracy.

## 5. Conclusions

In conclusion, artificial intelligence analyses provide highly effective results. However, these artificial neural network-based models are black-box models because the relational link between input and output is unobservable. We successfully combined artificial neural networks, machine learning, and conventional biomedinformatics to predict the overall survival outcome and molecular subtypes of DLBCL. This approach identified molecular targets that indicated poor and favorable survival in DLBCL in addition to showing that *MAPK3* correlated with the GCB subtype.

## Figures and Tables

**Table 1:** Multilayer perceptron analysis.

Dependent Variable	OS Outcome	Molecular Subtype (GCB, ABC, Unspecified)	Molecular Subtype (GCB, ABC+Unspecified)
Case processing			
Training set	72/105 (68.6%)	77/104 (74.0%)	76/104 (73.1%)
Testing set	33/105 (31.4%)	27/106 (26.0%)	28/104 (26.9%)
Input layer			
Covariates	730	730	730
Units	730	730	730
Rescaling	Standardized	Standardized	Standardized
Hidden Layer			
Number	1	1	1
Units	6	11	14
Activation function	Hyperbolic tangent	Hyperbolic tangent	Hyperbolic tangent
Output layer			
Num. Dependent variables	1	1	1
Units	2	3	2
Activation function	Softmax	Softmax	Softmax
Error function	Cross-entropy	Cross-entropy	Cross-entropy
Model summary			
Training			
Cross-entropy error	27.884	5.061	0.594
Incorrect predictions %	15.3	1.3	<0.0001
Stopping rule used	1 consecutive step(s) with no decrease in error	1 consecutive step(s) with no decrease in error	1 consecutive step(s) with no decrease in error
Training time	00:01.4	00:01.1	00:00.6
Testing			
Cross-entropy error	13.847	12.155	2.662
Incorrect predictions %	18.20	18.5	3.6
Classification (% correct)			
Training	84.7	98.7	100
Testing	81.8	81.5	96.4
Area under the curve			
	0.898 (Alive)	0.995 (GCB)	1.0 (GCB)
	0.898 (Dead)	0.994 (ABC)	1.0 (ABC+Unspecified)
		0.989 (Unspecified)	

OS, overall survival; GCB, germinal center B-cell-like; ABC, activated B-cell-like (ABC). The molecular subtypes were based on the Lymph2Cx assay.

**Table 2:** Top 20 genes identified by the multilayer perceptron analysis.

	Overall Survival Outcome		Molecular Subtype			
	Dead/Alive		GCB/ABC/Unspecified		GCB/ABC+Unspecified	
Order	Gene	Normalized Importance	Gene	Normalized Importance	Gene	Normalized Importance
1	*CD55*	1.000	*ARG1*	1.000	*CD37*	1.000
2	*ARG1*	0.982	*CTAG1B*	0.959	*STAT6*	0.867
3	*SPANXB1*	0.949	*CD55*	0.950	*ATF2*	0.830
4	*CTAG1B*	0.946	*IL22*	0.915	*ROPN1*	0.819
5	*IFNA8*	0.853	*PRG2*	0.903	*C4B*	0.814
6	*CASP1*	0.851	*SSX1*	0.895	*NOTCH1*	0.805
7	*IL2*	0.834	*JAK3*	0.877	*CTAG1B*	0.797
8	*TNFSF12*	0.819	*PPBP*	0.869	*ICAM3*	0.796
9	*ANP32B*	0.795	*APP*	0.851	*CEACAM1*	0.783
10	*CTSG*	0.784	*TAL1*	0.839	*NOD2*	0.773
11	*THY1*	0.780	*IL4R*	0.831	*LAG3*	0.773
12	*REL*	0.779	*F12*	0.815	*GNLY*	0.767
13	*TLR2*	0.775	*MAP2K2*	0.810	*TP53*	0.762
14	*C8A*	0.767	*MAP4K2*	0.798	*CD46*	0.755
15	*IL22*	0.760	*MAPK14*	0.797	*MAPK3*	0.747
16	*TIRAP*	0.755	*A2M*	0.795	*TREM2*	0.739
17	*CCL15*	0.754	*LILRA4*	0.791	*REL*	0.736
18	*NRP1*	0.753	*MAPK1*	0.789	*NUP107*	0.722
19	*XCL2*	0.750	*DMBT1*	0.786	*IL17RB*	0.718
20	*MFGE8*	0.749	*NT5E*	0.781	*SPACA3*	0.714

The genes were ranked according to their normalized importance for predicting overall survival and molecular subtypes. The molecular subtypes were based on the Lymph2Cx assay.

**Table 3:** Correlations between the top 20 genes of the multilayer perceptron and the overall survival of the patients.

Gene	*Beta*	SE	Wald	Df	*p*	Hazard Risk	95.0% CI for HR	
							Lower	Upper
Step 1								
*CD55*	−0.06	0.29	0.04	1	0.851	0.9	0.5	1.7
*ARG1*	−0.54	0.22	6.00	1	0.014	0.6	0.4	0.9
*SPANXB1*	0.28	0.24	1.34	1	0.246	1.3	0.8	2.1
*CTAG1B*	−0.02	0.17	0.01	1	0.903	1.0	0.7	1.4
*IFNA8*	0.47	0.26	3.22	1	0.073	1.6	1.0	2.7
*CASP1*	1.42	0.37	14.51	1	0.000	4.1	2.0	8.6
*IL2*	−0.04	0.28	0.03	1	0.874	1.0	0.6	1.7
*TNFSF12*	−1.00	0.37	7.28	1	0.007	0.4	0.2	0.8
*ANP32B*	0.06	0.53	0.01	1	0.907	1.1	0.4	3.0
*CTSG*	0.45	0.14	10.11	1	0.001	1.6	1.2	2.1
*THY1*	0.42	0.32	1.74	1	0.188	1.5	0.8	2.9
*REL*	−0.32	0.25	1.60	1	0.205	0.7	0.4	1.2
*TLR2*	0.51	0.29	3.13	1	0.077	1.7	0.9	2.9
*C8A*	−0.18	0.33	0.29	1	0.593	0.8	0.4	1.6
*IL22*	−0.10	0.17	0.31	1	0.580	0.9	0.6	1.3
*TIRAP*	−0.32	0.39	0.69	1	0.406	0.7	0.3	1.6
*CCL15*	−0.17	0.33	0.29	1	0.593	0.8	0.4	1.6
*NRP1*	−0.78	0.35	5.07	1	0.024	0.5	0.2	0.9
*XCL2*	−0.09	0.16	0.31	1	0.579	0.9	0.7	1.3
*MFGE8*	−0.04	0.30	0.02	1	0.888	1.0	0.5	1.7
Step 14 (last)								
*ARG1*	−0.46	0.19	5.84	1	0.016	0.6	0.4	0.9
*IFNA8*	0.37	0.20	3.64	1	0.056	1.5	1.0	2.1
*CASP1*	1.34	0.31	18.59	1	0.000	3.8	2.1	7.1
*TNFSF12*	−0.78	0.27	8.42	1	0.004	0.5	0.3	0.8
*CTSG*	0.37	0.13	8.00	1	0.005	1.4	1.1	1.9
*REL*	−0.33	0.19	3.20	1	0.074	0.7	0.5	1.0
*NRP1*	−0.53	0.28	3.67	1	0.055	0.6	0.3	1.0

Multivariate Cox regression analysis for overall survival; backward conditional.

**Table 4:** Machine learning analysis for predicting the overall survival outcome and molecular subtypes.

Dependent Variable (Target Variable)	Model	Overall Accuracy (%)	Num. of Fields (Genes)
Overall survival outcome (Dead/Alive)	Logistic regression	100	730
	Discriminant	100	730
	SVM	100	730
	CHAID tree	97.1	10
	C5 tree	96.2	12
	C&R tree	86.7	36
	Neural net.	69.6	730
	KNN algorithm	60.9	730
	Bayesian net.	26.4	730
Molecular subtype (GCB/ABC/Unspecified)	Logistic regression	100	730
	Discriminant	100	730
	SVM	100	730
	C5 tree	96.2	7
	CHAID tree	96.2	8
	Neural net.	92.3	730
	Quest	75.9	12
	C&R tree	75	6
	KNN algorithm	71.2	730
	Bayesian net.	0.9	730
Molecular subtype (GCB/ABC+Unspecified)	Logistic regression	100	730
	Discriminant	100	730
	SVM	100	730
	Neural net.	99	730
	CHAID tree	99.1	6
	C5 tree	97.1	5
	KNN algorithm	84.6	730
	Quest	83.6	6
	C&R tree	50.9	730
	Bayesian net.	0	730

**Table 5:** Associations between the top 20 genes identified by the multilayer perceptron and molecular subtypes (GCB, ABC+Unspecified).

Gene	*Beta*	SE	Wald	df	*p*	Odds Ratio (OR)	95.0% CI for OR	
							Lower	Upper
Step 1								
*CD37*	1.59	0.52	9.41	1	0.002	4.9	1.8	13.4
*STAT6*	−2.50	0.93	7.20	1	0.007	0.1	0.0	0.5
*ATF2*	0.02	1.27	0.00	1	0.990	1.0	0.1	12.2
*ROPN1*	0.33	0.49	0.45	1	0.503	1.4	0.5	3.6
*C4B*	−0.15	0.34	0.20	1	0.654	0.9	0.4	1.7
*NOTCH1*	−0.42	0.80	0.27	1	0.605	0.7	0.1	3.2
*CTAG1B*	0.46	0.62	0.56	1	0.453	1.6	0.5	5.3
*ICAM3*	0.07	0.53	0.02	1	0.897	1.1	0.4	3.0
*CEACAM1*	0.30	0.28	1.12	1	0.291	1.3	0.8	2.4
*NOD2*	−0.01	0.42	0.00	1	0.990	1.0	0.4	2.3
*LAG3*	0.61	0.40	2.32	1	0.128	1.8	0.8	4.0
*GNLY*	0.78	0.34	5.35	1	0.021	2.2	1.1	4.2
*TP53*	−0.93	0.65	2.01	1	0.156	0.4	0.1	1.4
*CD46*	1.04	0.65	2.57	1	0.109	2.8	0.8	10.1
*MAPK3*	−0.87	1.05	0.70	1	0.404	0.4	0.1	3.2
*TREM2*	−0.82	0.42	3.77	1	0.052	0.4	0.2	1.0
*REL*	−0.96	0.55	3.10	1	0.079	0.4	0.1	1.1
*NUP107*	0.59	1.03	0.33	1	0.568	1.8	0.2	13.7
*IL17RB*	0.31	0.19	2.70	1	0.100	1.4	0.9	2.0
*SPACA3*	−0.23	0.45	0.25	1	0.618	0.8	0.3	1.9
*Constant*	6.82	3.86	3.12	1	0.077	917.1		
Step 14 (last)								
*CD37*	1.05	0.37	8.17	1	0.004	2.9	1.4	5.9
*STAT6*	−1.96	0.75	6.76	1	0.009	0.1	0.0	0.6
*GNLY*	1.01	0.25	15.70	1	0.000	2.7	1.7	4.5
*CD46*	0.78	0.46	2.86	1	0.091	2.2	0.9	5.4
*TREM2*	−0.55	0.33	2.76	1	0.097	0.6	0.3	1.1
*REL*	−1.13	0.49	5.28	1	0.022	0.3	0.1	0.8
*IL17RB*	0.38	0.15	6.35	1	0.012	1.5	1.1	2.0
*Constant*	4.67	2.53	3.41	1	0.065	107.0		

Multivariate binary logistic regression for molecular subtypes (GCB vs. ABC+Unclassified), backward conditional. In the regression analysis, the GCB is the reference group.

## Data Availability

The source codes and data from Tokai University presented in this study are available on reasonable request to the corresponding author (J.C.). The raw gene expression data are not publicly available because of a data protection policy for patient data.

## Supplementary Materials

The list of housekeeping genes and logistic regression are available online at https://www.mdpi.com/article/10.3390/cancers13246384/s1.

## Appendix A

**Table A1:** Correlations between the top 20 genes of the multilayer perceptron and the overall survival of the patients (univariate analysis).

Order	Gene	*Beta*	SE	Wald	df	*p*	Hazard Risk	95.0% CI for HR	
								Lower	Upper
1	*CD55*	−0.12	0.22	0.31	1	0.577	0.9	0.6	1.4
2	*ARG1*	0.12	0.09	1.56	1	0.211	1.1	0.9	1.3
3	*SPANXB1*	0.14	0.13	1.15	1	0.283	1.2	0.9	1.5
4	*CTAG1B*	0.06	0.12	0.22	1	0.641	1.1	0.8	1.3
5	*IFNA8*	0.17	0.14	1.49	1	0.222	1.2	0.9	1.5
6	*CASP1*	0.45	0.20	5.24	1	0.022	1.6	1.1	2.3
7	*IL2*	0.04	0.15	0.09	1	0.767	1.0	0.8	1.4
8	*TNFSF12*	−0.05	0.16	0.11	1	0.74	0.9	0.7	1.3
9	*ANP32B*	0.06	0.31	0.04	1	0.851	1.1	0.6	1.9
10	*CTSG*	0.10	0.06	2.75	1	0.097	1.1	1.0	1.3
11	*THY1*	−0.08	0.14	0.30	1	0.586	0.9	0.7	1.2
12	*REL*	−0.40	0.20	3.83	1	0.05	0.7	0.5	1.0
13	*TLR2*	0.24	0.16	2.20	1	0.138	1.3	0.9	1.7
14	*C8A*	0.06	0.19	0.10	1	0.752	1.1	0.7	1.6
15	*IL22*	0.06	0.13	0.17	1	0.677	1.1	0.8	1.4
16	*TIRAP*	−0.01	0.28	0.00	1	0.967	1.0	0.6	1.7
17	*CCL15*	−0.03	0.22	0.02	1	0.901	1.0	0.6	1.5
18	*NRP1*	−0.08	0.18	0.19	1	0.663	0.9	0.7	1.3
19	*XCL2*	0.12	0.12	0.97	1	0.324	1.1	0.9	1.4
20	*MFGE8*	−0.11	0.13	0.68	1	0.41	0.9	0.7	1.2

Univariate Cox regression analysis for overall survival. Each gene was analyzed individually as a quantitative variable.

**Table A2:** Correlations between the top 20 genes of the multilayer perceptron and the molecular subtypes GCB vs. ABC+Unspecified (univariate analysis).

Order	Gene	*Beta*	SE	Wald	df	*p*	Hazard Risk	95.0% CI for HR	
								Lower	Upper
1	*CD37*	0.16	0.19	0.67	1	0.413	1.2	0.8	1.7
2	*STAT6*	−0.84	0.48	3.07	1	0.080	0.4	0.2	1.1
3	*ATF2*	−0.64	0.65	0.96	1	0.327	0.5	0.1	1.9
4	*ROPN1*	0.47	0.33	2.09	1	0.149	1.6	0.8	3.0
5	*C4B*	0.22	0.14	2.65	1	0.103	1.2	1.0	1.6
6	*NOTCH1*	−0.18	0.32	0.32	1	0.575	0.8	0.4	1.6
7	*CTAG1B*	0.28	0.24	1.32	1	0.250	1.3	0.8	2.1
8	*ICAM3*	0.44	0.28	2.55	1	0.111	1.6	0.9	2.7
9	*CEACAM1*	0.38	0.18	4.36	1	0.037	1.5	1.0	2.1
10	*NOD2*	0.27	0.18	2.26	1	0.133	1.3	0.9	1.9
11	*LAG3*	0.35	0.14	6.22	1	0.013	1.4	1.1	1.9
12	*GNLY*	0.62	0.17	13.10	1	0.000	1.9	1.3	2.6
13	*TP53*	−0.97	0.39	6.02	1	0.014	0.4	0.2	0.8
14	*CD46*	0.39	0.34	1.30	1	0.254	1.5	0.8	2.9
15	*MAPK3*	−0.49	0.49	0.99	1	0.319	0.6	0.2	1.6
16	*TREM2*	−0.19	0.22	0.76	1	0.383	0.8	0.5	1.3
17	*REL*	−1.16	0.36	10.61	1	0.001	0.3	0.2	0.6
18	*NUP107*	−0.59	0.58	1.04	1	0.309	0.6	0.2	1.7
19	*IL17RB*	0.17	0.10	2.94	1	0.087	1.2	1.0	1.5
20	*SPACA3*	0.14	0.22	0.39	1	0.530	1.1	0.7	1.8

Univariate binary logistic regression analysis for molecular subtype. Each gene was analyzed individually as a quantitative variable (GCB as reference).

**Table A3:** Annotations of the overall survival top genes.

Order	Gene	Gene Class	Immune Response Category	Annotation
1	*CD55*	Immune Response	N/A	CD molecules, innate immune response
2	*ARG1*	Immune Response	N/A	Response to drug
3	*SPANXB1*	CT Antigen	N/A	N/A
4	*CTAG1B*	CT Antigen	N/A	N/A
5	*IFNA8*	Immune Response	Interleukins	Innate immune response, interleukins
6	*CASP1*	Immune Response	N/A	Innate immune response
7	*IL2*	Immune Response	Cytokines, T-Cell Functions, Regulation	Adaptive immune response, anti-inflammatory cytokines, B-cell activation, cytokines and receptors, innate immune response, interleukins, Th1 and Th2 differentiation, T-cell differentiation, T-cell polarization, T-cell regulators
8	*TNFSF12 (TWEAK)*	Immune Response	Chemokines, TNF Superfamily	Chemokines and receptors, TNF superfamily members and their receptors
9	*ANP32B (APRIL)*	Immune Response—Cell Type Specific (Th)	Cell Functions	Basic cell functions, cell type specific
10	*CTSG*	Immune Response—Cell Type Specific (Mast cell)	Regulation, Pathogen Defense	Cell type specific, defense response to fungus, positive regulation of immune response
11	*THY1*	Immune Response	N/A	CD molecules
12	*REL*	Immune Response	Regulation	Transcription factors
13	*TLR2*	Immune Response	TLR	CD molecules, innate immune response, toll-like receptor
14	*C8A*	Immune Response	Complement	Complement pathway, innate immune response
15	*IL22*	Immune Response	Cytokines	Acute-phase response, anti-inflammatory cytokines, interleukins
16	*TIRAP*	Immune Response	N/A	Innate immune response
17	*CCL15*	Immune Response	Chemokines	Adaptive immune response, chemokines and receptors, inflammatory response
18	*NRP1*	Immune Response	Cell Functions	Basic cell functions, CD molecules
19	*XCL2*	Immune Response	Chemokines	Chemokines and receptors
20	*MFGE8*	Immune Response	Transporter Functions	Receptors involved in phagocytosis

N/A, not applicable; Th, T helper; TLR, toll-like receptor; CD, cluster differentiation.

**Table A4:** Annotations of the molecular subtypes top genes.

Order	Gene	Gene Class	Immune Response Category	Annotation
1	*CD37*	Immune Response	N/A	Adaptive immune response, CD molecules
2	*STAT6*	Immune Response—Cell Type Specific (Th2 cell)	Chemokines, Regulation, T-Cell Functions	Adaptive immune response, cytokines and receptors, cell type specific, Th2 orientation, transcription factors, transcriptional regulators
3	*ATF2*	Immune Response	N/A	Innate immune response
4	*ROPN1*	CT Antigen	N/A	N/A
5	*C4B*	Immune Response	Complement	Complement pathway, innate immune response
6	*NOTCH1*	Immune Response	Regulation	Transcriptional regulators
7	*CTAG1B*	CT Antigen	N/A	N/A
8	*ICAM3*	Immune Response	Adhesion, Regulation	Adhesion, CD molecules, regulation of immune response
9	*CEACAM1*	Immune Response	Adhesion	Adhesion, CD molecules
10	*NOD2*	Immune Response	Cytokines	Innate immune response, cytokines and receptors
11	*LAG3*	Immune Response—Checkpoint	Regulation, T-Cell Functions	Adaptive immune response, CD molecules, negative regulation of immune response, T-cell activation
12	*GNLY*	Immune Response—Cell Type Specific (Cytotoxic cells)	Cell Functions, Cytotoxicity	Adaptive immune response, basic cell functions, cell type specific, cytotoxicity
13	*TP53*	Immune Response	T-Cell Functions	T-cell proliferation
14	*CD46*	Immune Response	N/A	CD molecules, innate immune response
15	*MAPK3*	Immune Response	N/A	Innate immune response
16	*TREM2*	Immune Response	N/A	Humoral immune response
17	*REL*	Immune Response	Regulation	Transcription factors
18	*NUP107*	Immune Response—Cell Type Specific (Th cell)	Cell Cycle	Cell type specific, M phase of mitotic cell cycle
19	*IL17RB*	Immune Response	Chemokines	Chemokines and receptors
20	*SPACA3*	CT Antigen	N/A	N/A

N/A, not applicable; Th, T helper; TLR, toll-like receptor; CD, cluster differentiation.

**Table A5:** Characteristics of the series of DLBCL used in the immunohistochemical analysis.

Variable	Frequency (%)	Univariate Cox Regression for Overall Survival			
		*p*	Hazard R.	Lower	Upper
Male	54/97 (55.7)	0.941	1	0.5	1.9
Age > 60	67/97 (69.1)	0.004	4	1.6	10.3
Ann Arbor stage III–IV	42/89 (47.2)	0.06	1.9	0.9	3.7
ECOG performance status ≥2	13/78 (16.7)	0.0002	4.3	1.9	9.4
Serum LDH high (>219)	58/96 (60.4)	0.004	3.1	1.4	6.8
Extranodal sites >1	18/73 (24.7)	0.003	3.1	1.5	6.4
IPI					
Low	31/81 (38.3)	Reference	-	-	-
Low–intermediate	25/81 (30.9)	0.008	3.7	1.4	9.8
High–intermediate	14/81 (17.3)	0.033	3.3	1.1	9.9
High	11/81 (13.6)	0.004	5.3	1.7	16.5
sIL2R high (>530)	70/91 (76.9)	0.017	4.2	1.3	13.7
B symptoms	19/80 (23.8)	0.395	1.4	0.7	3
Location					
Nodal (+spleen)	53/97 (54.6)	Reference	-	-	-
Waldeyer’s ring	9/97 (9.3)	0.167	0.2	0	1.8
Gastrointestinal	10/97 (10.3)	0.748	0.8	0.2	2.8
Other extranodal	25/97 (25.8)	0.216	1.5	0.8	2.9
Treatment					
RCHOP	65/91 (71.4)	Reference	-	-	-
RCHOP-like	20/91 (22.0)	0.136	1.7	0.8	3.6
Others	6/91 (6.6)	0.133	2.5	0.8	8.5
Response to treatment					
CR	64/86 (74.4)	Reference	-	-	-
PD	11/86 (12.8)	6.5 × 10^−11^	26.3	9.8	70.2
PR	11/86 (12.8)	1.7 × 10^−8^	12.7	5.3	30.9
Epstein–Barr virus (EBER+)	12/95 (15.8)	0.004	3	1.4	6.4
Hans classifier					
GCB	31/95 (32.6)	Reference	-	-	-
Non-GCB	64/95 (67.4)	0.013	2.8	1.3	6.4
Immune phenotype					
CD3+	0/97 (0)	N/A	-	-	-
CD5+	14/96 (14.6)	0.736	0.9	0.4	2.1
CD20+	93/97 (95.9)	0.417	0.6	0.1	2.3
CD10+	29/96 (30.2)	0.011	0.3	0.1	0.8
MUM1+ (IRF4)	76/96 (79.2)	0.193	1.7	0.8	3.9
BCL2+	76/96 (79.2)	0.054	2.8	0.9	7.8
BCL6+	64/96 (66.7)	0.821	0.9	0.5	1.8
RGS1 high (>3%)	51/95 (53.7)	0.013	2.5	1.2	5.2
Molecular analysis					
*MYD88* L265P mutation	3/39 (7.7)	0.542	0.5	0.1	4
*BCL2* translocation	2/42 (4.8)	0.993	0.9	0.1	7.4
*MYC* translocation	7/46 (15.2)	0.814	0.9	0.3	2.9
*BCL2*/*MYC* double hit	1/42 (2.4)	0.321	2.8	0.4	21.6
