# Supplementary material for: Artificial Neural Networks Predicted the Overall Survival and Molecular Subtypes of Diffuse Large B-Cell Lymphoma Using a Pancancer Immune-Oncology Panel
Source: Cancers (Basel). 2021 Dec 20;13(24):6384. doi: 10.3390/cancers13246384 (PMC8699516; doi:10.3390/cancers13246384)
Supplement: Supplementary file 1 [file cancers-13-06384-s001.zip › cancers-1470601-supplementary.pdf]

Supplementary Material

List of housekeeping genes of the PanCancer Immune Profiling panel

1. *ABCF1*
2. *AGK*
3. *ALAS1*
4. *AMMECR1L*
5. *CC2D1B*
6. *CNOT10*
7. *CNOT4*
8. *COG7*
9. *DDX50*
10. *DHX16*
11. *DNAJC14*
12. *EDC3*
13. *EIF2B4*
14. *ERCC3*
15. *FCF1*
16. *G6PD*
17. *GPATCH3*
18. *GUSB*
19. *HDAC3*
20. *HPRT1*
21. *MRPS5*
22. *MTMR14*
23. *NOL7*
24. *NUBP1*
25. *POLR2A*
26. *PPIA*
27. *PRPF38A*
28. *SAP130*
29. *SDHA*
30. *SF3A3*
31. *TBP*
32. *TLK2*
33. *TMUB2*

34. *TRIM39*
35. *TUBB*
36. *USP39*
37. *ZC3H14*
38. *ZKSCAN5*
39. *ZNF143*
40. *ZNF346*

<u>Logistic regression</u>

1. Equation dead overall survival outcome:

-100.7 * A2M +
-60.67 * ABCB1 +
-107.3 * ABL1 +
-2.179 * ADA +
-12.71 * ADORA2A +
39.28 * AICDA +
54.68 * AIRE +
-65.65 * AKT3 +
26.36 * ALCAM +
79.26 * AMBP +
42.25 * AMICA1 +
158.1 * ANP32B +
54.17 * ANXA1 +
29.06 * APOE +
19.04 * APP +
-21.68 * ARG1 +
-115.5 * ARG2 +
-95.3 * ATF1 +
11.53 * ATF2 +
-92.44 * ATG10 +
60.91 * ATG12 +
252.4 * ATG16L1 +
-115.1 * ATG5 +
-9.984 * ATG7 +
42.73 * ATM +

-25.74 * AXL +

56.87 * BAGE +

-35.01 * BATF +

-47.85 * BAX +

-236.0 * BCL10 +

54.57 * BCL2 +

-18.24 * BCL2L1 +

-2.471 * BCL6 +

33.2 * BID +

-105.8 * BIRC5 +

-0.6957 * BLK +

-48.86 * BLNK +

28.65 * BMI1 +

32.92 * BST1 +

30.19 * BST2 +

-123.1 * BTK +

-8.602 * BTLA +

9.881 * C1QA +

21.54 * C1QB +

109.7 * C1QBP +

-12.9 * C1R +

150.3 * C1S +

-19.62 * C2 +

65.52 * C3 +

20.26 * C3AR1 +

-55.13 * C4B +

50.77 * C4BPA +

-8.163 * C5 +

-10.14 * C6 +

9.298 * C7 +

-9.457 * C8A +

83.46 * C8B +

-94.95 * C8G +

20.22 * C9 +

-44.28 * CAMP +

27.56 * CARD11 +

-105.5 * CARD9 +

63.47 * CASP1 +

65.38 * CASP10 +

114.9 * CASP3 +

-142.3 * CASP8 +

-64.38 * CCL1 +

-37.1 * CCL11 +

-17.69 * CCL13 +

4.887 * CCL14 +

-9.589 * CCL15 +

127.2 * CCL16 +

10.07 * CCL17 +

-31.86 * CCL18 +

-65.17 * CCL19 +

56.45 * CCL2 +

-5.474 * CCL20 +

-50.11 * CCL21 +

28.17 * CCL22 +

0.1598 * CCL23 +

26.5 * CCL24 +

77.66 * CCL25 +

21.0 * CCL26 +

-49.97 * CCL27 +

-89.75 * CCL28 +

157.7 * CCL3 +

-325.9 * CCL3L1 +

253.0 * CCL4 +

-120.0 * CCL5 +

-70.36 * CCL7 +

-31.38 * CCL8 +

-123.3 * CCND3 +

5.925 * CCR1 +

30.56 * CCR2 +

7.664 * CCR3 +

6.176 * CCR4 +

-27.38 * CCR5 +

27.87 * CCR6 +

-34.18 * CCR7 +

16.58 * CCR9 +

98.48 * CCRL2 +

-80.83 * CD14 +

47.14 * CD160 +

19.1 * CD163 +

+ 1099.0

2. Logistic regression for molecular subtype

Equation for ABC subtype:

-179.7 * A2M +

-219.5 * ABCB1 +

-290.6 * ABL1 +

-266.5 * ADA +

-234.8 * ADORA2A +

157.2 * AICDA +

87.06 * AIRE +

1.88 * AKT3 +

-38.95 * ALCAM +

579.5 * AMBP +

285.0 * AMICA1 +

1156.7 * ANP32B +

305.8 * ANXA1 +

-37.53 * APOE +

-541.6 * APP +

-309.2 * ARG1 +

-81.69 * ARG2 +

-733.7 * ATF1 +

102.2 * ATF2 +

-822.8 * ATG10 +

249.9 * ATG12 +

576.2 * ATG16L1 +

-393.7 * ATG5 +

287.4 * ATG7 +

396.8 * ATM +

447.7 * AXL +

-325.0 * BAGE +

-131.8 * BATF +

726.6 * BAX +

-827.6 * BCL10 +

-28.64 * BCL2 +

-141.6 * BCL2L1 +

-103.8 * BCL6 +

269.0 * BID +

60.69 * BIRC5 +

-142.3 * BLK +

-121.8 * BLNK +

761.9 * BMI1 +

18.37 * BST1 +

489.5 * BST2 +

-32.64 * BTK +

-63.68 * BTLA +

620.4 * C1QA +

-231.0 * C1QB +

-456.3 * C1QBP +

-857.7 * C1R +

1065.3 * C1S +

-1031.0 * C2 +

207.3 * C3 +

298.2 * C3AR1 +

-53.56 * C4B +

627.3 * C4BPA +

-110.9 * C5 +

29.37 * C6 +

-26.44 * C7 +

-495.5 * C8A +

148.0 * C8B +

-494.1 * C8G +

302.2 * C9 +

-91.05 * CAMP +

-67.44 * CARD11 +

-236.6 * CARD9 +

394.5 * CASP1 +

-302.3 * CASP10 +

-345.3 * CASP3 +

-464.5 * CASP8 +

-177.0 * CCL1 +

-19.48 * CCL11 +

-158.2 * CCL13 +

-64.83 * CCL14 +

-47.47 * CCL15 +

592.1 * CCL16 +

132.4 * CCL17 +

-155.2 * CCL18 +

-270.4 * CCL19 +

333.4 * CCL2 +

31.72 * CCL20 +

-38.49 * CCL21 +

73.09 * CCL22 +

423.8 * CCL23 +

-132.4 * CCL24 +

-42.2 * CCL25 +

28.45 * CCL26 +

-213.9 * CCL27 +

-329.9 * CCL28 +

643.5 * CCL3 +

-1312.9 * CCL3L1 +

1235.5 * CCL4 +

-527.8 * CCL5 +

64.86 * CCL7 +

67.21 * CCL8 +

-86.9 * CCND3 +

-294.3 * CCR1 +

354.1 * CCR2 +

209.7 * CCR3 +

-3.327 * CCR4 +

-239.2 * CCR5 +

-53.42 * CCR6 +

-246.1 * CCR7 +

32.14 * CCR9 +

-104.9 * CCRL2 +

-0.7647 * CD14 +

75.37 * CD160 +

+ 1691.5

Equation For Unspecified

-112.7 * A2M +

-134.9 * ABCB1 +

-165.3 * ABL1 +

-141.5 * ADA +

-126.9 * ADORA2A +

89.61 * AICDA +

31.16 * AIRE +

-5.653 * AKT3 +

-25.63 * ALCAM +

307.9 * AMBP +

160.3 * AMICA1 +

633.7 * ANP32B +

158.1 * ANXA1 +

-24.92 * APOE +

-268.3 * APP +

-162.0 * ARG1 +

-50.57 * ARG2 +

-419.9 * ATF1 +

75.38 * ATF2 +

-440.6 * ATG10 +

151.3 * ATG12 +

339.6 * ATG16L1 +

-226.1 * ATG5 +

128.5 * ATG7 +

229.7 * ATM +

247.1 * AXL +

-171.4 * BAGE +

-77.18 * BATF +

362.5 * BAX +

-476.6 * BCL10 +

-5.718 * BCL2 +

-83.2 * BCL2L1 +

-54.88 * BCL6 +

148.0 * BID +

23.99 * BIRC5 +

-75.49 * BLK +

-62.61 * BLNK +

412.5 * BMI1 +

15.79 * BST1 +

276.6 * BST2 +

-39.21 * BTK +

-33.51 * BTLA +

311.9 * C1QA +

-102.7 * C1QB +

-222.3 * C1QBP +

-442.8 * C1R +

555.5 * C1S +

-547.6 * C2 +

116.3 * C3 +

178.3 * C3AR1 +

-39.22 * C4B +

340.1 * C4BPA +

-51.02 * C5 +

17.51 * C6 +

-16.45 * C7 +

-271.0 * C8A +

102.0 * C8B +

-270.9 * C8G +

144.0 * C9 +

-49.41 * CAMP +

-24.22 * CARD11 +

-137.6 * CARD9 +

216.3 * CASP1 +

-152.5 * CASP10 +

-160.3 * CASP3 +

-268.0 * CASP8 +

-98.1 * CCL1 +

-7.844 * CCL11 +

-83.4 * CCL13 +

-30.89 * CCL14 +

-18.98 * CCL15 +

329.7 * CCL16 +

72.76 * CCL17 +

-80.73 * CCL18 +

-144.0 * CCL19 +

187.9 * CCL2 +

15.88 * CCL20 +

-19.65 * CCL21 +

43.97 * CCL22 +

218.8 * CCL23 +

-77.53 * CCL24 +

-11.89 * CCL25 +

21.61 * CCL26 +

-117.6 * CCL27 +

-179.1 * CCL28 +

365.7 * CCL3 +

-740.3 * CCL3L1 +

685.8 * CCL4 +

-291.6 * CCL5 +

26.67 * CCL7 +

16.13 * CCL8 +

-61.09 * CCND3 +

-132.3 * CCR1 +

181.8 * CCR2 +

118.1 * CCR3 +

-5.648 * CCR4 +

-115.5 * CCR5 +

-20.19 * CCR6 +

-134.3 * CCR7 +

19.09 * CCR9 +
-30.23 * CCRL2 +
-34.59 * CD14 +
42.79 * CD160 +
+ 1196.5

3. Logistic regression molecular subtype 2

Equation for ABC+Unspecified

-199.5 * A2M +
-241.8 * ABCB1 +
-311.4 * ABL1 +
-278.9 * ADA +
-247.3 * ADORA2A +
168.6 * AICDA +
81.2 * AIRE +
-2.406 * AKT3 +
-44.02 * ALCAM +
606.5 * AMBP +
304.1 * AMICA1 +
1223.4 * ANP32B +
317.2 * ANXA1 +
-42.56 * APOE +
-554.1 * APP +
-322.1 * ARG1 +
-90.22 * ARG2 +
-787.8 * ATF1 +
120.8 * ATF2 +
-863.5 * ATG10 +
273.8 * ATG12 +
625.1 * ATG16L1 +
-423.3 * ATG5 +
284.9 * ATG7 +
427.8 * ATM +
474.7 * AXL +
-339.3 * BAGE +
-142.6 * BATF +
745.0 * BAX +

-890.6 * BCL10 +

-23.73 * BCL2 +

-153.5 * BCL2L1 +

-108.5 * BCL6 +

284.9 * BID +

58.1 * BIRC5 +

-148.9 * BLK +

-126.1 * BLNK +

802.6 * BMI1 +

23.2 * BST1 +

523.3 * BST2 +

-48.55 * BTK +

-66.45 * BTLA +

637.7 * C1QA +

-228.6 * C1QB +

-464.3 * C1QBP +

-889.4 * C1R +

1108.2 * C1S +

-1079.1 * C2 +

221.0 * C3 +

325.2 * C3AR1 +

-63.14 * C4B +

661.1 * C4BPA +

-110.9 * C5 +

32.0 * C6 +

-29.25 * C7 +

-523.8 * C8A +

170.3 * C8B +

-522.7 * C8G +

305.4 * C9 +

-95.99 * CAMP +

-62.96 * CARD11 +

-255.5 * CARD9 +

417.4 * CASP1 +

-311.1 * CASP10 +

-346.2 * CASP3 +

-500.2 * CASP8 +
-187.9 * CCL1 +
-18.74 * CCL11 +
-165.1 * CCL13 +
-65.52 * CCL14 +
-45.58 * CCL15 +
629.7 * CCL16 +
140.2 * CCL17 +
-161.3 * CCL18 +
-283.2 * CCL19 +
356.1 * CCL2 +
32.56 * CCL20 +
-39.76 * CCL21 +
79.89 * CCL22 +
439.4 * CCL23 +
-143.3 * CCL24 +
-37.25 * CCL25 +
34.05 * CCL26 +
-226.5 * CCL27 +
-347.8 * CCL28 +
689.3 * CCL3 +
-1402.4 * CCL3L1 +
1312.6 * CCL4 +
-559.8 * CCL5 +
62.77 * CCL7 +
57.48 * CCL8 +
-100.8 * CCND3 +
-292.2 * CCR1 +
366.5 * CCR2 +
223.9 * CCR3 +
-6.035 * CCR4 +
-242.8 * CCR5 +
-50.53 * CCR6 +
-260.0 * CCR7 +
34.97 * CCR9 +
-93.01 * CCRL2 +

-23.28 * CD14 +

80.7 * CD160 +

+ 1973.6
